# Identification and Characterization of an Antennae-Specific Glutathione S-Transferase From the Indian Meal Moth

**DOI:** 10.3389/fphys.2021.727619

**Published:** 2021-08-26

**Authors:** Hongmin Liu, Yin Tang, Qinying Wang, Hongzhong Shi, Jian Yin, Chengjun Li

**Affiliations:** ^1^College of Agronomy, Xinyang Agriculture and Forestry University, Xinyang, China; ^2^College of plant protection, Hebei Agricultural University, Baoding, China; ^3^Tobacco Research Institute, Henan Academy of Agricultural Sciences, Xuchang, China

**Keywords:** Plodia interpunctella, glutathione S-transferases, pheromone, volatile, semiochemicals, degradation, enzyme

## Abstract

Insect glutathione-S-transferases (GSTs) play essential roles in metabolizing endogenous and exogenous compounds. GSTs that are uniquely expressed in antennae are assumed to function as scavengers of pheromones and host volatiles in the odorant detection system. Based on this assumption, antennae-specific GSTs have been identified and functionally characterized in increasing number of insect species. In the present study, 17 putative GSTs were identified from the antennal transcriptomic dataset of the Indian meal moth, *Plodia interpunctella*, a severe stored-grain pest worldwide. Among the GSTs, only PiGSTd1 is antennae-specific according to both Fragments Per Kilobase Million (FPKM) and quantitative real-time PCR (qRT-PCR) analysis. Sequence analysis revealed that PiGSTd1 has a similar identity as many delta GSTs from other moths. Enzyme kinetic assays using 1-chloro-2,4-dinitrobenzene (CDNB) as substrates showed that the recombinant PiGSTd1 gave a *K*_*m*_ of 0.2292 ± 0.01805 mM and a *V*_*max*_ of 14.02 ± 0.2545 μmol·mg^−1^·min^−1^ under the optimal catalytic conditions (35°C and pH = 7.5). Further analysis revealed that the recombinant PiGSTd1 could efficiently degrade the sex pheromone component Z9-12:Ac (75.63 ± 5.52%), as well as aldehyde volatiles, including hexanal (89.10 ± 2.21%), heptanal (63.19 ± 5.36%), (*E*)-2-octenal (73.58 ± 3.92%), (*E*)-2-nonenal (75.81 ± 1.90%), and (*E*)-2-decenal (61.13 ± 5.24%). Taken together, our findings suggest that PiGSTd1 may play essential roles in degrading and inactivating a variety of odorants, especially sex pheromones and host volatiles of *P. interpunctella*.

## Introduction

Glutathione S-transferases (GSTs, EC 2.5.1.18) exist ubiquitously in various organisms (Enayati et al., 2005). As a family of multifunctional detoxification enzymes, GSTs play vital roles in metabolizing a wide range of endogenous and exogenous compounds as well as in degrading them into less-toxic metabolites by catalyzing the conjugation of electrophilic molecules with glutathione (GSH) (Singh et al., 2001; Huang et al., 2017). It is widely accepted that GSTs exert their detoxification function *via* two domains: One is the highly conserved N-terminal GSH binding domain (G-site) and the other is the C-terminal hydrophobic substrate binding domain (H-site) (Enayati et al., 2005). Insect GSTs are classified into the cytosolic, microsomal, and mitochondrial subgroups based on their cellular locations (Hayes et al., 2015). The majority of insect cytosolic GSTs are divided into six subclasses (i.e., delta, epsilon, omega, sigma, theta, and zeta) mainly according to their sequence identities, genomic structures, and biochemical properties (Sheehan et al., 2001; Yu et al., 2008). Among these subclasses, only delta and epsilon are considered insect-specific, while others are found in a variety of invertebrates and vertebrates (Labade et al., 2018).

During the past two decades, an increasing number of studies have focused on the crucial roles of insect GSTs in the detoxification of harmful stimuli, such as phytochemicals and insecticides (Glaser et al., 2013; Liu et al., 2015; Zou et al., 2016). NlGST1-1 from *Nilaparvata lugens* can detoxify various plant metabolites so this planthopper can rapidly adapt to a broader host range (Sun et al., 2013). SlGSTE1 in the gut of *Spodoptera litura* and HaGST-8 from *Helicoverpa armigera* show higher binding activity to insecticides like chlorpyrifos, deltamethrin, malathion, phoxim, and dichloro-diphenyl-trichloroethane (DDT), resulting in insecticide resistance in pests (Xu et al., 2015; Labade et al., 2018). Besides the functions of metabolism and detoxification, antennae-specific GSTs can also function as odorant-degrading enzymes (ODEs) as part of the olfactory system. During the process of odor recognition, antennal GSTs can quickly remove or degrade the odorants from olfactory receptors (ORs) to maintain sensitivity and fidelity of the chemoreceptor (Vogt and Riddiford, 1981; Younus et al., 2014; Durand et al., 2018). BmGSTd4, an antennae-specific GST in the male silk moth, plays a dual role in the detoxification of xenobiotic compounds and the signal termination of sex pheromone signals (Tan et al., 2014). GST-msolf1 from antennal sensilla of *Manduca sexta* can modify (*E*)-2-hexenal, suggesting that the GST is involved in inactivating host plant volatiles (Rogers et al., 1999). Hence, our study on antennae-specific GSTs could deepen understanding of insect olfactory recognition and contribute to the subsequent development of potential pest control strategies.

The Indian meal moth, *Plodia interpunctella* (Lepidoptera: Pyraloidea, Pyralidae), a cosmopolitan stored-product pest, causes severe economic loss yearly (Mohandass et al., 2007). The sex pheromone-based monitoring approach has been proven accurate and efficient in monitoring populations of *P. interpunctella* (Campos and Phillips, 2014). Therefore, revealing the mechanism of pheromone recognition could benefit the development of novel attractants or repellents against this pest. Recently, Jia et al. (2018) have identified a series of odorant-related proteins and chemoreceptors from the antennae of *P*. *interpunctella* through transcriptomic sequencing. More recently, we reported that antennal-specific carboxylesterases of *P*. *interpunctella* (PintCXEs) respond to sex pheromone and environmental volatiles (Liu et al., 2019). However, whether GSTs are involved in pheromone recognition remains unknown.

This study aimed to identify GSTs from *P*. *interpunctella* antennae, analyze their sequences, and evaluate the characteristics of antennae-specific PiGSTs in degrading sex pheromone and host volatiles. Our results will provide fundamental information on the GSTs in the antennae of *P. interpunctella* and pave the way for further research on the semiochemical-based control of this pest.

## Materials and Methods

### Insects and Tissue RNA Collection

*P. interpunctella* were reared on crushed wheat seeds in the laboratory of the Plant Protection Institute, Hebei Academy of Agricultural and Forestry Sciences, at 28 ± 1°C, 60 ± 5% RH and 14:10 L:D photoperiod (Jia et al., 2018). The last-instar larvae were separated and individually reared in glass vials (diameter 2 cm, height 4.5 cm) until their eclosion. The samples from tissues (antennae, thoraces, abdomens, legs, and wings) were prepared following our previous method (Liu et al., 2019). All samples were immediately frozen in liquid nitrogen and stored at −80°C until further RNA extraction. Total RNA extraction, purity evaluation, and concentration determination were performed as previously reported (Jia et al., 2018).

### Identification of GST Genes

The identification of antennal GSTs from *P*. *interpunctella* was mainly based on previously reported transcriptome datasets (accession number: SRR6002827 and SRR6002828) (Jia et al., 2018). The putative GSTs were preliminarily retrieved from annotations based on the latest database, including non-redundant protein (NR), Gene Ontology (GO), Swiss-Prot, and the Kyoto Encyclopedia of Genes and Genomes (KEGG). Subsequently, all candidates were manually validated using the NCBI BLASTx (http://blast.ncbi.nlm.nih.gov/) with an E-value of < 10^−5^.

### Expression of GST Genes Using Quantitative Real-Time PCR (qRT-PCR)

Quantitative real-time PCR tests were conducted on an ABI 7500 (Thermo Fisher Scientific, United States) using Bestar^®^ SybrGreen qPCR mastermix kit (DBI^®^ Bioscience) and using the β-actin gene, which was identified from the antennal transcriptome of *P. interpunctella*, as the reference gene (paired primers: 5′-GTATCAACGGATTTGGTCG-3′ and 5′-CACCTTCCAAGTGAGCAGAT-3′) (Liu et al., 2019). Each reaction was completed in a 20 μL system blend, comprising 10 μL of Bestar SyBr Green qPCR mastermix, 0.2 μM of each primer, 0.4 μL of 50x ROX Reference Dye, 2 μL of cDNA template, and 6.8 μL of RNase-free water at conditions of 1 cycle of 95°C for 2 min, followed by 40 cycles of 95°C for 10 s, 55°C for 34 s, and 72°C for 30 s. Each sample had three independent biological replicates, and each replicate was tested in three technical repeats. All primers are available in Supplementary Table 1. The amplification efficiency for each primer pair ranged from 91.6% to 100.3% based on the standard curve analysis. Relative expression of all GST genes was determined using the comparative 2^−ΔΔCt^ method (Livak and Schmittgen, 2001). The heatmaps were created by Heatmapper (http://www.heatmapper.ca/) based on the transformed data of log_2_ (2^−ΔΔCt^ + 1) values (Babicki et al., 2016).

### Bioinformatics Analyses

The GST sequences were characterized by corresponding bioinformatics tools. GST-ORFs were identified using ORF Finder (http://www.ncbi.nlm.nih.gov/gorf/gorf.html). The sequence lengths, molecular weights (MWs), and isoelectric points (pI) were predicted by using ExPASy tools (https://web.expasy.org/compute_pi/) (Gasteiger et al., 2005). The conserved domains were predicted by using the hmmsearch tool from the pfam website (http://pfam.xfam.org/) (Mistry et al., 2021). Identification of conserved motifs of GSTs was conducted with the MEME online program for protein sequence (http://meme.nbcr.net/meme/intro.html) (Bailey et al., 2009) with the optimized parameters being any number of repetitions, a maximum number of 10 motifs, and optimum 6–50 residue length per motif.

### Phylogenetic Construction

Deduced amino acid sequences of GST genes from different insects were aligned with the GST sequence identified from the antennae of *P. interpunctella* using ClustalW with default parameters (https://www.genome.jp/tools-bin/clustalw). After sequence alignments, the phylogenetic tree was constructed by MEGA5.0 software using the neighbor-joining method with the following parameters: Poisson model, pairwise deletion, and 1,000 bootstrap replications (Tamura et al., 2011). The dendrogram was further decorated using Evolview software (https://www.evolgenius.info/evolview/). The homologous GST sequences were used to reconstruct a phylogenetic tree from eight species, including *Plutella xylostella* (You et al., 2015), *Cydia pomonella* (Huang et al., 2017), *Bombyx mori* (Yu et al., 2008), *Chilo suppressalis* (Liu et al., 2015), *Acyrthosiphon pisum* (Francis et al., 2001), *Drosophila melanogaster* (Younus et al., 2014), *Anopheles gambiae* (Ding et al., 2003), and *Tribolium castaneum* (Shi et al., 2012). All sequences were obtained from NCBI (https://www.ncbi.nlm.nih.gov/).

### Homology Modeling of PiGSTd1

The homology model was constructed by the SWISS-MODEL server (https://swissmodel.expasy.org/interactive). The models of PiGSTd1 were built based on the target-template alignment using ProMod3 (Guex et al., 2009). The QMEAN scoring function was used to assess the global and per-residue model quality (Studer et al., 2020). Then, an automated model BmGSTd1 (PDB ID: 4e8e.1) was selected as the template from PDB database. Pictures of three-dimensional structures were generated with PyMOL (DeLano, 2002). Multisequence alignments were performed using ClustalX 2.1, and the results were presented by GeneDoc software (http://nrbsc.org/gfx/genedoc). The secondary structure was predicted with PSIPRED software (McGuffin et al., 2000).

### PiGSTd1 Plasmid Construction, Expression, and Purification

The PiGSTd1 sequence without signal peptide was amplified by PCR using TransStart^®^ FastPfu PCR SuperMix (TransGen Biotech, China). The paired primers were forward 5′-ATGCCGGCTCAAGCCATCAA-3′ and reverse 5′-CTAATCTTTCTTCAGAAATGATGC-3′. The amplification was carried out under the conditions of denaturation at 95°C for 1 min followed by 35 cycles of 95°C for 20 s, 55°C for 20 s, and 72 °C for 1 min, and a final extension at 72°C for 5 min. The PCR products were ligated into a pEASY-Blunt E1 Expression vector (TransGen Biotech, China) and transformed into *Escherichia coli Trans-T1* (Liu et al., 2017). After sequence confirmation by Sangon Biotechnology (Shanghai, China), the positive recombinant plasmids were designated as pEASY-Blunt E1-PiGSTd1.

PiGSTd1 expression and purification were conducted as previously described with a slight modification (Song et al., 2020). Briefly, the recombinant vector (pEASY-Blunt E1-PiGSTd1) was transformed into *E. coli* BL21 (DE3), and the positive clones were isolated for expression. Cultures were started from single colonies, in LB broth with 50 μg/mL ampicillin in a 37°C shaker (220 rpm). When OD of 600 nm reached 0.6, isopropyl *β*-D-1-thiogalactopyranoside (IPTG) was added to 1 mM. After cultured for 6 h at 25°C and 220 rpm, cells were harvested by centrifugation at 8,000 g at 4°C and suspended in 20 ml of PBS buffer (pH = 7.0).

After ultrasonic cell disintegration, the collected bacteria were centrifuged at 14,000 rpm at 4°C for 20 min. After confirming the expression by 12% SDS–PAGE, the supernatants were loaded on a Ni-chelating affinity column (GE, United States), which had been equilibrated with 20 mM Tris–HCl (pH = 7.9) supplemented with 100 mM NaCl, and eluted with imidazole (50, 100, 150, and 200 mM) in an ascending series. The recombinant PiGSTd1 purity was assayed by SDS–PAGE. Its concentration was determined using Bradford's method with BCA Protein Assay Kits (Legend biotech, Beijing, China). Proteins were stored at −20°C before use.

### Kinetic Properties of PGSTd1

The kinetic parameter of PiGSTd1 was determined based on the CDNB (1-chloro-2,4-dinitrobenzene) method (Li et al., 2018). Briefly, 0.4 μg of the purified PiGSTd1 was added into 200 μL acetate–phosphate buffer (pH 7.5) containing 50 mM GSH and a series of CDNB (0, 0.2, 0.4, 0.6, 0.8, 1.0, 1.2, and 1.4 mM) at 35°C in a transparent 96-well plates, and the absorbance at 340 nm in 0–1 min was recorded in a Multiskan Spectrum Microplate Spectrophotometer (BioTek, Shoreline, WA). Heat-inactivated PiGSTd1 was used as the negative control. The *Km* and *Vmax* were calculated by the linear regression of a double reciprocal plot (Balakrishnan et al., 2018). To optimize the reaction pH and temperature of PiGSTd1, the assays were conducted at fixed concentrations of GSH (1 mM) and CDNB (0.5 mM) with varying acetate–phosphate buffer (pH = 5.5, 6.0, 6.5, 7.0, 7.5, 8.0, 8.5 and 9.0) and reaction temperature (20, 25, 30, 35, 40, 50, 55, and 60°C for 30 min). All determinations were performed three times.

### Enzymatic Degradation Tests of Recombinant PiGSTd1

A GC-MS (7890A-5975C; Agilent, United States) with a DB-WAX column (30m × 0.25mm × 0.25μm, Agilent) was used to evaluate the degradation activities of PiGSTd1 on the main sex pheromone and environmental volatiles (Supplementary Table 2). The degradation assays were conducted in 1 mL acetate–phosphate buffer (pH = 7.0) containing 2.5 μg purified PiGSTd1, 10 mM GSH, and 20 μg substrates. After reacting for 1 h at 35°C, the reaction mixture was extracted with 1 mL hexane immediately. Subsequently, substrates in the organic phase were qualitatively and quantitatively analyzed on the GC-MS with the chromatographic conditions setting as helium carrier gas at 1 ml·min^−1^; oven temperature initiated at 50°C (hold 1 min), increased to 120°C at 5°C·min^−1^ (hold 2 min), and subsequently increased to 230°C at 10°C·min^−1^ (hold 5 min). The ionization current and ionization voltage were 100 μA and 70 eV, respectively. All assays were repeated three times with the heat-inactivated PiGSTd1 as the negative control. Degradation data were analyzed by one-way ANOVA (SPSS 19.0 for Windows) with Tukey's test. The least significant difference was set at *P* < 0.05.

## Results

### Identification and Classification of PiGSTs

From the antennal transcriptome of *P. interpunctella*, we identified a total of 17 sequences encoding putative GSTs, which were designated as PiGSTd1-PiGSTm3. Sequence characteristics (ORFs, MW, and pI) and Blastx results are listed in Table 1. Among all PiGSTs, 15 sequences were intact ORFs, while PiGSTo4 and PiGSTd2 were incomplete with truncated 3′-regions. The sequence lengths of the PiGSTs ranged from 149 to 290 amino acid (aa), and their calculated MWs ranged from 16.35 to 33.15 kDa. BLAST_X_ search of the best hits showed that all PiGST sequences shared relatively high sequence identities (62−97%) with their respective orthologs from other lepidopteran species (Table 1).

**Table 1:** Details of the 17 GSTs identified in *Plodia interpunctella* antennae.

**Clade**	**Gene Name**	**GenBank accession**	**Full Length**	**ORF(aa)**	**pI**	**MW(Da)**	**Blastx annotation (Name/Species)**	**Accession number**	**Score**	**E-value**	**Identity**
Delta	PiGSTd1	MZ410553	Y	245	5.15	27761.04	Glutathione S-transferase delta 1 [*Chilo suppressalis*]	AKS40338.1	379	3E-131	73%
	PiGSTd2	MZ410560	N	237	-	-	Glutathione S-transferase delta 1 [*Aphis gossypii*]	AFM78644.1	444	3E-157	89%
	PiGSTd3	MZ410545	Y	215	6.91	24098.6	Glutathione S-transferase delta [*Antheraea pernyi*]	ACB36909.1	399	3E-140	89%
Epsilon	PiGSTe1	MZ410556	Y	228	6.76	25835.97	Glutathione S-transferase 1 [*Papilio xuthus*]	KPJ03136.1	312	2E-105	63%
	PiGSTe2	MZ410551	Y	217	5.29	24549.23	Glutathione S-transferase GSTD1 [*Helicoverpa armigera*]	AIB07715.1	330	7E-113	73%
Omega	PiGSTo1	MZ410557	Y	256	6.15	29124.28	Glutathione S-transferase [*Plutella xylostella*]	AHW45906.1	436	3E-153	79%
	PiGSTo2	MZ410559	Y	290	8.39	33155.47	Glutathione S-transferase omega 2 [*Bombyx mori*]	ABD36306.1	348	1E-117	56%
	PiGSTo3	MZ410550	Y	242	7.64	27990.27	Glutathione S-transferase omega 3 [*Cnaphalocrocis medinalis*]	AIZ46903.1	365	6E-126	70%
	PiGSTo4	MZ410558	N	241	-	-	Glutathione S-transferase gst [*Trifolium pratense*]	PNX77761.1	376	2E-130	80%
Sigma	PiGSTs1	MZ410548	Y	206	6.34	23737.23	Glutathione S-transferase sigma 4 [*Cnaphalocrocis medinalis*]	AIZ46904.1	277	3E-92	64%
	PiGSTs2	MZ410552	Y	205	6.35	23572.19	Glutathione S-transferase sigma-1 [*Cydia pomonella*]	ARM39007.1	318	2E-108	70%
Theta	PiGSTt1	MZ410547	Y	232	8.8	27123.11	Glutathione S-transferase theta-1 [*Helicoverpa armigera*]	XP_021200219.1	341	1E-116	68%
Zeta	PiGSTz1	MZ410546	Y	215	8.06	24615.57	Glutathione S-transferase zeta-1 [*Cydia pomonella*]	ARM39005.1	432	2E-153	97%
Unclassified	PiGSTu1	MZ410554	Y	234	6.23	26630.45	Glutathione S-transferase 1-1 [*Papilio polytes*]	NP_001298693.1	400	6E-140	79%
Microsomal	PiGSTm1	MZ410561	Y	154	9.55	17032.02	Microsomal glutathione S-transferase [*Antheraea yamamai*]	AII16887.1	214	4E-69	69%
	PiGSTm2	MZ410555	Y	149	9.98	16654.6	Microsomal glutathione S-transferase 1-1 [*Spodoptera litura*]	AIH07603.1	186	6E-58	62%
	PiGSTm3	MZ410549	Y	149	9.77	16357.2	Microsomal glutathione transferase [*Heliothis virescens*]	ADH16761.1	232	3E-76	74%

### Phylogenetic Tree Analysis

**Figure 1:**
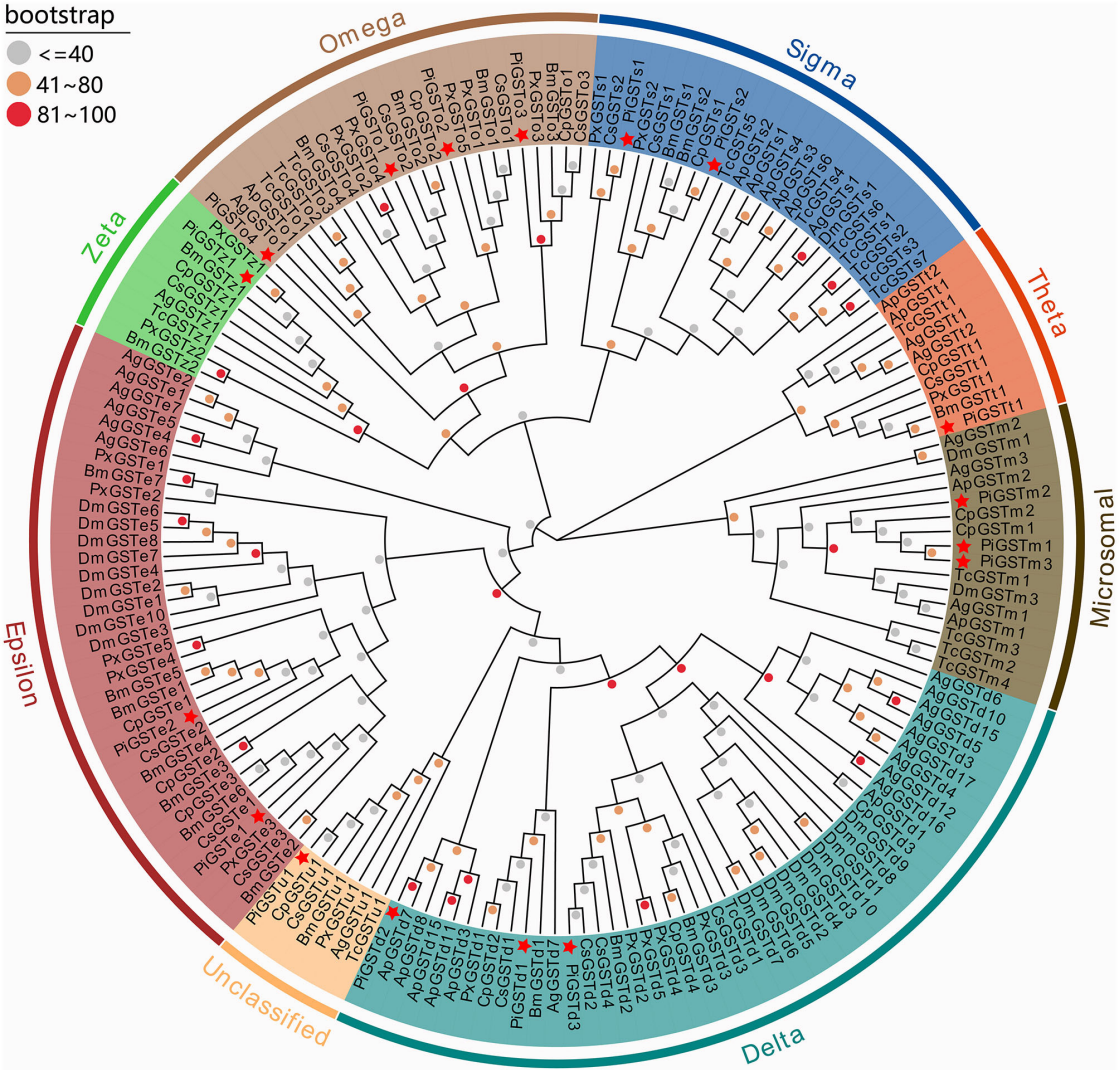
Phylogenetic tree of GSTs from nine insect species. Eight GST branches are distinguished with different background color, and PiGSTs were marked with red stars. Dots with different colors present different bootstrap values: gray, ≤40; orange, 40–80; red, 81–100. Ag, *Anopheles gambiae*; Ap, *Acyrthosiphon pisum*; Bm, *Bombyx mori*; Cs, *Chilo suppressalis*; Cp, *Cydia pomonella*; Dm, *Drosophila melanogaster*; Pi, *Plodia interpunctella*; Px, *Plutella xylostella*; Tc, *Tribolium castaneum*.

The phylogenetic tree was reconstructed with 169 GST sequences from nine species, including model insects (e.g., *B. mori* and *D. melanogaster*), typical species in varying families, as well as congeneric Pyraloid moths. Although these GSTs were derived from diverse species, they showed relative conservation in classification. According to their sequence similarities, 17 PiGSTs were distributed into eight branches of the phylogenetic tree: delta (PiGSTd1 to PiGSTd3), epsilon (PiGSTe1 and PiGSTe2), omega (PiGSTo2 to PiGSTo4), sigma (PiGSTs1 and PiGSTs2), theta (PiGSTt1), zeta (PiGSTz1), and unclassified class (PiGSTm1) (Figure 1). PiGSTd1 was clustered with CpGSTd2, a well-characterized enzyme involved in odorant degradation for chemosensory perception in *C. pomonella* (Huang et al., 2017), indicating it could potentially degrade odorants.

### Conserved Domains and Motif Composition Analysis of PiGSTs

**Figure 2:**
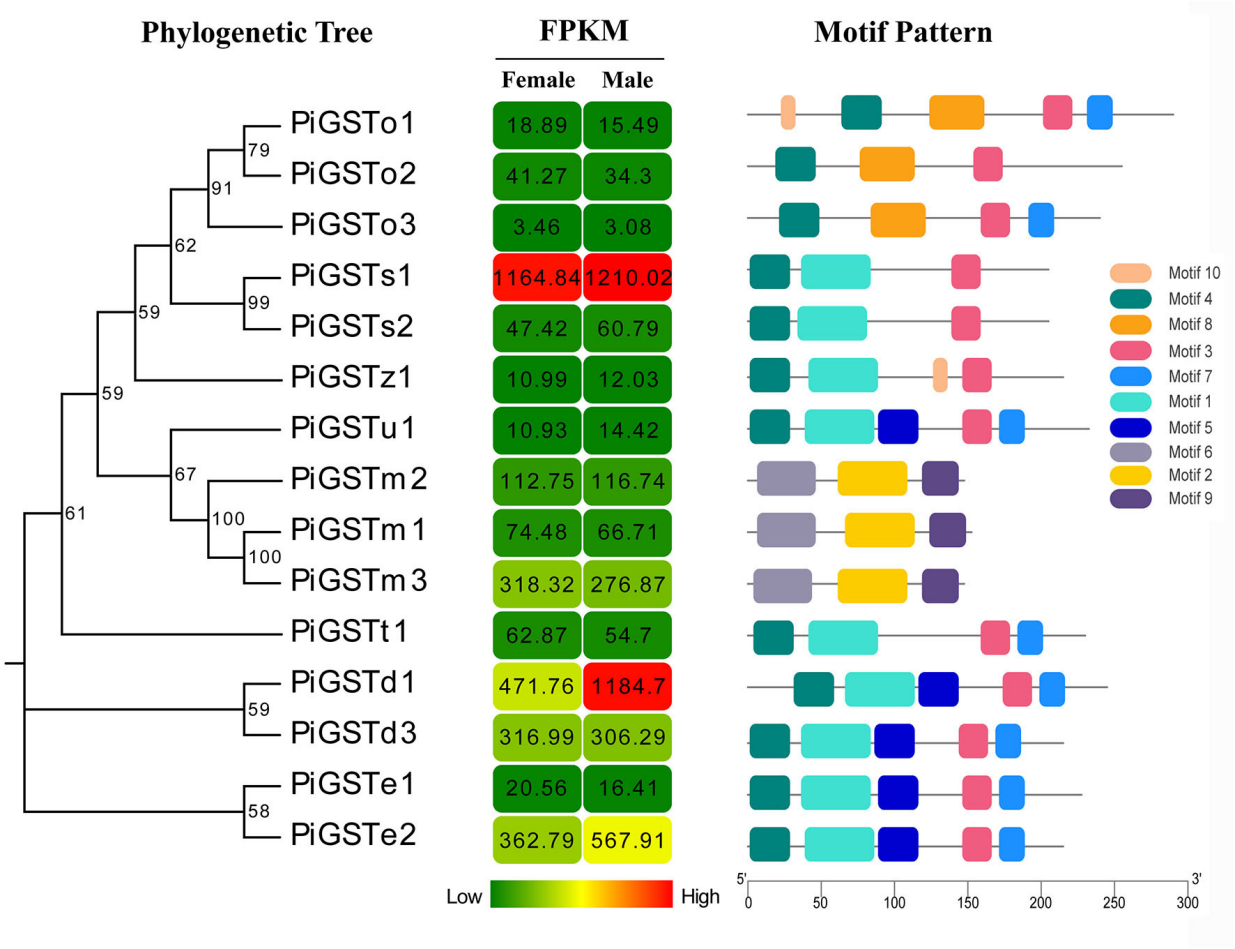
Phylogenetic relationships, FPKM of PiGSTs, and architecture of the conserved motif patterns. The phylogenetic tree was constructed based on the full-length sequences of PiGSTs using MEGA 5.0 software. The sequence information for each motif is provided in Supplementary Figure 2. The conserved motifs are displayed in colored boxes, and the length of protein can be estimated using the scale at the bottom.

The analyses of conserved domains among the PiGSTs revealed two domains of the protein sequences: a fairly conserved N-terminal domain and a more variable C-terminal domain among different subclasses (Supplementary Figure 1). Besides, a member of conserved membrane-associated proteins was identified in eicosanoid and glutathione metabolism (MAPGE) from microsomal GSTs (Supplementary Figure 1). A schematic representing the structure of all complete PiGSTs sequences was constructed from the MEME motif analysis results. PiGSTs in the same subclass usually shared a similar motif composition and showed highly similar motif distributions, e.g., the clustered PiGST pairs, PiGSTs1-2 and PiGSTe12 (Figure 2). Among all motifs, motif 3 and motif 4 were found in all cytosolic GST proteins, while motif 2, motif 6, and motif 9 were exclusively expressed in microsomal GSTs (PiGSTm1-3).

### Tissue Expression Profile of PiGSTs

**Figure 3:**
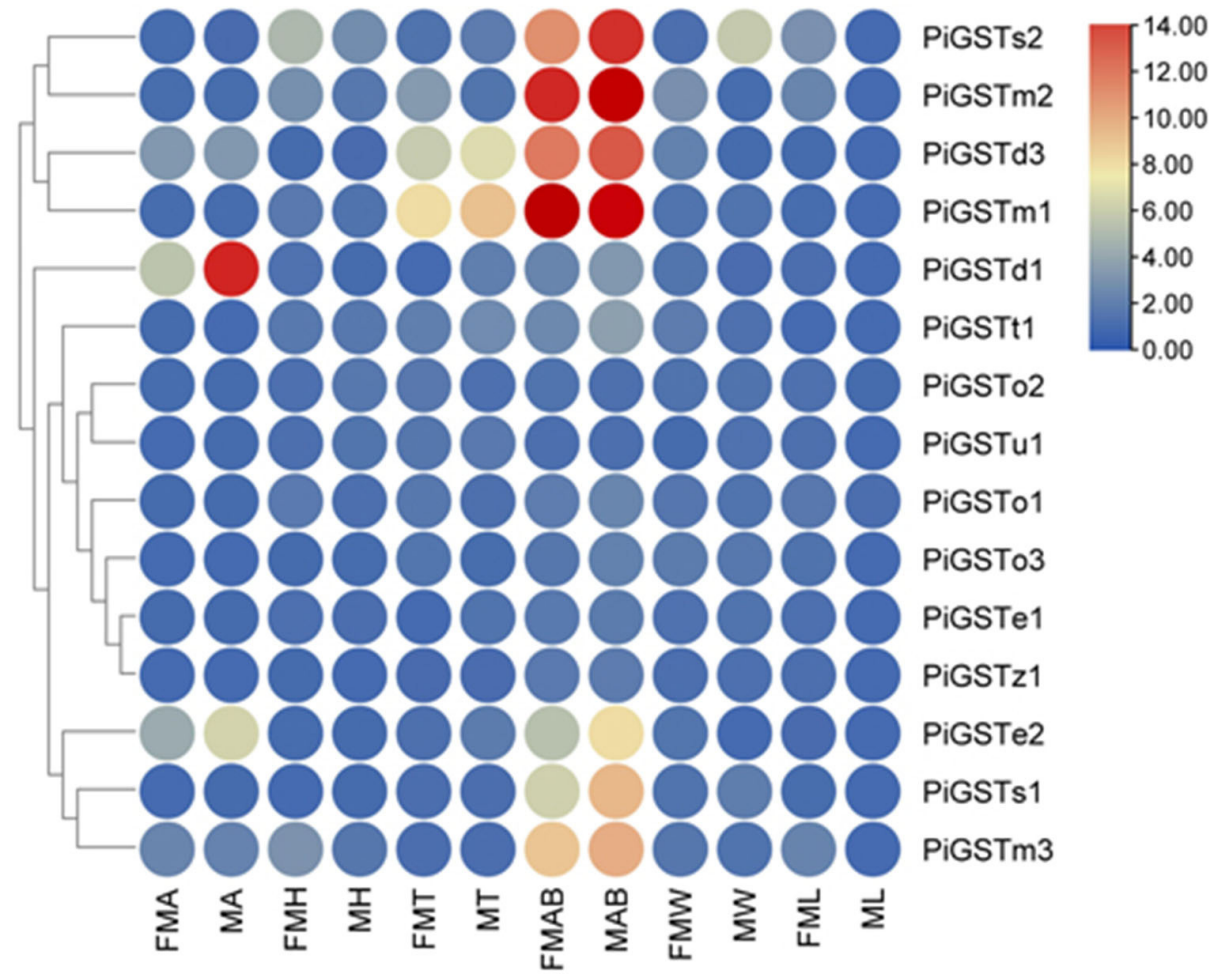
Tissue expression pattern of the PiGST Genes. Levels of gene expression were normalized relative to that in the leg (one-fold). FMA, female antennae; MA, male antennae; FMH, female heads; MH, male heads; FMT, female thoraxes; MT, male thoraxes; FMAB, female abdomens; MAB, male abdomens; FMW, female wings; MW, male wings; FML, female legs; ML, male legs.

Based on qRT-PCR determination, only PiGSTd1 expression was antennae-specific, and its expression level was significantly higher in males than in females (Figure 3), indicating that it has a close association with odorant recognition. In contrast, PiGSTe2 was almost equally expressed in female and male antennae and was also found in the abdomens, but it was not antennae-enriched. PiGSTo2, PiGSTo3, PiGSTm1, PiGSTm2, PiGSTm3, PiGSTs1, PiGSTs2, PiGSTz1, PiGSTe1, PiGSTd3, and PiGSTt1 were abundantly expressed in the abdomens of both sexes (Figure 3). Other GST genes were ubiquitously expressed in all tested tissues.

### Sequence Analysis of PiGSTd1

**Figure 4:**
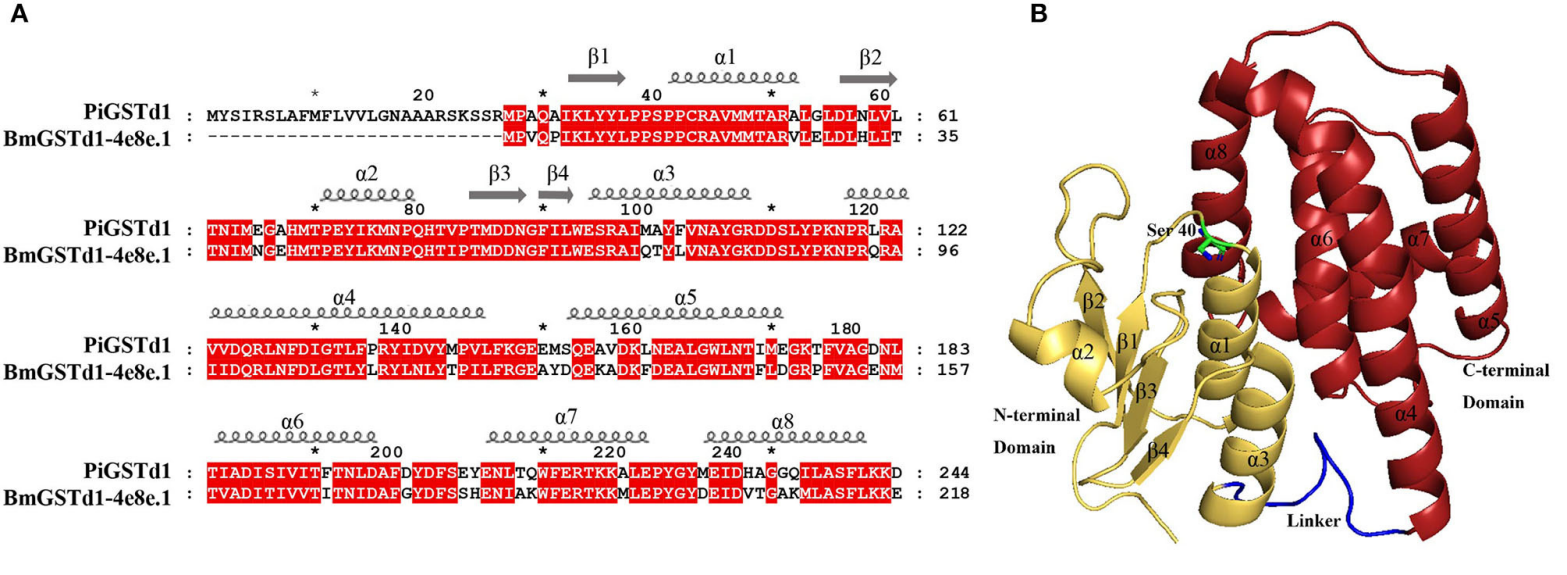
Structural characterization of PiGSTd1. **(A)** Sequence alignment of PiGSTd1 and BmGSTd1 (PDB ID: 4e8e.1). **(B)** Homology model of PiGSTd1. Yellow, red, and blue represent N-terminal domain, C-terminal domain, and linker, respectively. Ser40 in green is proposed to be catalytically essential.

According to a multiple alignment of PiGSTd1 with delta GSTs from other moths, PiGSTd1 showed relatively high identities (63.11–68.83%) with HvGSTd1 (AWX68884.1), OfGSTd1 (QIC35737.1), CsGSTd1 (AKS40338.1), SeGSTd1 (ASN63930.1), BmGSTd1 (NP_001037183.1), and PrGSTd1 (APW77568.1) (Supplementary Figure 3), indicating high conservation between PiGSTd1 and moth delta GSTs. Additionally, the multiple alignments and homology modeling on the basis of BmGSTd1 suggested that PiGSTd1 adopted the classic GST fold and was composed of an *N*-terminal domain, a *C*-terminal domain, and a linker in between (Figure 4). In the conserved *N*-terminal domain, a three α-helices and four β-strands motif (β*αβαββα*) of thioredoxin fold-served as the glutathione binding site (G-site). Ser40 in PiGSTd1 appeared to be responsible for enzyme catalysis.

### Enzymatic Properties of PiGSTd1

**Figure 5:**
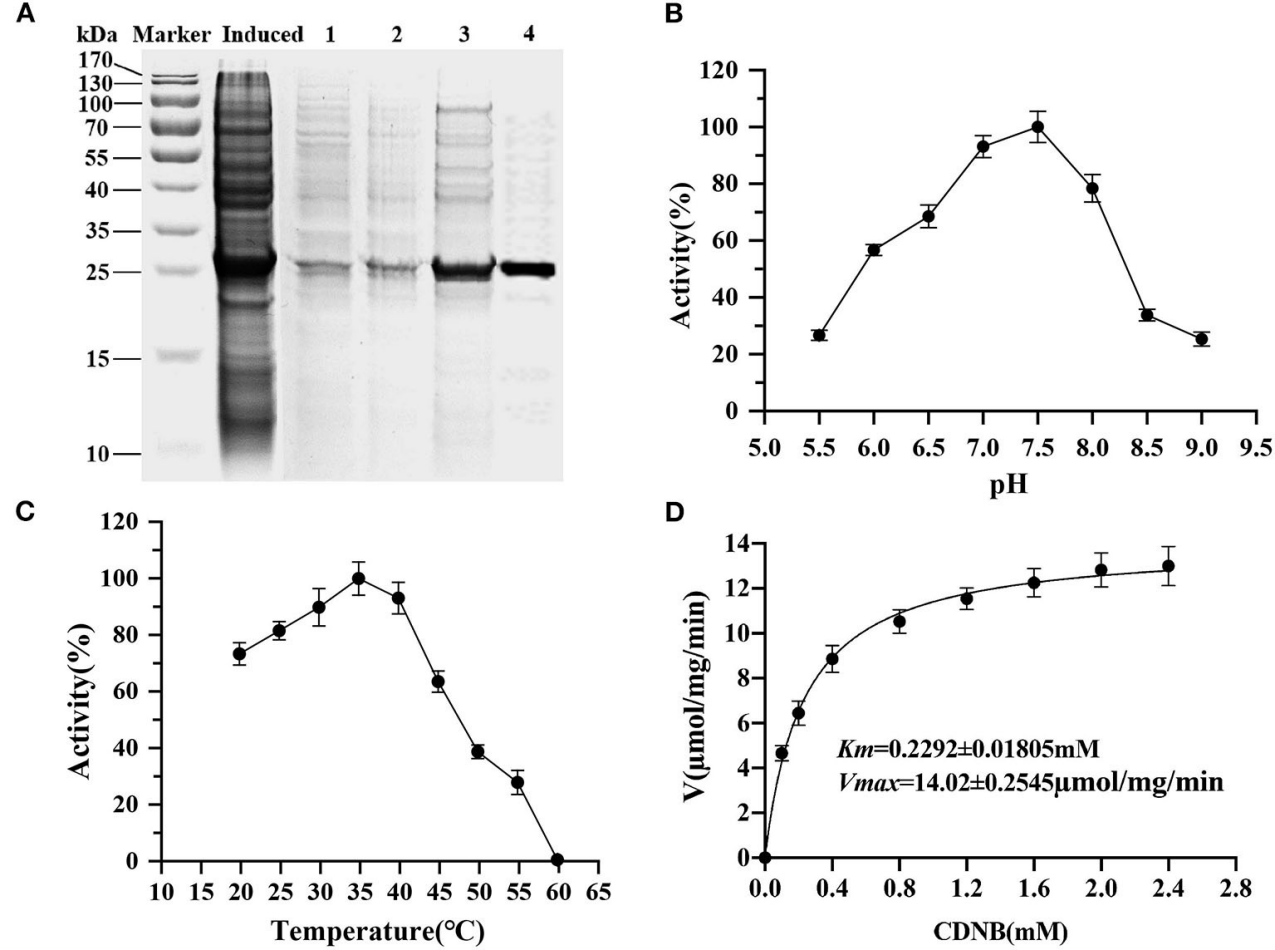
Kinetic properties of recombinant PiGSTd1. **(A)** Purification of recombinant pEasy-Blunt-E1-PiGSTd1. Induced: the crude extracts from the bacterial pellets with 1mM isopropyl β-D-1-thiogalactopyranoside (IPTG) induction. 1–4: samples eluted with binding buffers containing 50, 100, 150, and 200mM imidazole, respectively. **(B)** Enzyme kinetic of PiGSTd1 with different CDNB concentrations and a fixed GSH concentration. **(C)** Optimal pH of PiGSTd1 assayed using 100mM acetate-phosphate buffer at varying pH. **(D)** The catalytic activity of PiGSTd1 was determined by preincubating enzyme solution at different temperatures.

The entire coding sequence of PiGSTd1 was successfully expressed in *E. coli* strain BL21 through pEASY-Blunt E1 vector. SDS–PAGE showed that Ni^+^-column-purified PiGSTd1 displayed a single band with a MW of ~27kDa (Figure 5A). Using CDNB and reduced GSH as substrates, the optimized catalytic conditions for PiGSTd1 were 35 °C and pH=7.0 (Figures 5B,C). Under these conditions, *K*_*m*_ and *V*_*max*_ of recombinant PiGSTd1 were determined as 0.2292 ± 0.01805mM and 14.02±0.2545 μmol·mg^−1^·min^−1^, respectively (Figure 5D).

### *In vitro* Degradation Ability of Recombinant PiGSTd1

The ability of recombinant PiGSTd1 to degrade odorants was evaluated by GC-MS. The results showed that PiGSTd1 more efficiently degraded the sex pheromone component Z9-12:Ac (75.63 ± 5.52%) as compared with the pheromone analog Z8-12:Ac (58.47 ± 1.64%), despite only a differently positioned double bond. Besides sex pheromones, PiGSTd1 also displayed high efficiency in degrading various host odorants and environmental volatiles (Supplementary Table 2), e.g., α-pinene (68.83 ± 2.37%), hexanal (89.10 ± 2.21%), heptanal (63.19 ± 5.36%), (*E*)-2-octenal (73.58 ± 3.92%), (*E*)-2-nonenal (75.81 ± 1.90%), and (*E*)-2-decenal (61.13 ± 5.24%). The results indicated that PiGSTd1 highly expressed in *P. interpunctella* antennae was involved in degrading sex pheromones and host volatiles.

## Discussion

Insect antennal-specific GSTs play important roles in metabolizing a wide range of endogenous and exogenous compounds, including plant secondary compounds, insecticides, and odorant molecules (Huang et al., 2017). Therefore, deciphering the role of insect antennal GSTs will greatly extend our knowledge of the insect olfactory system. In the present study, we identified 17 PiGST genes from the antennal transcriptome of *P*. *interpunctella*, which is more than the number identified from the antennae of *C. suppressalis* (16 genes) (Liu et al., 2015) and *C. pomonella* (10 genes) (Huang et al., 2017), but fewer than the number in other insects, for example, *S. littoralis* (33 genes) (Legeai et al., 2011) and *D. melanogaster* (31 genes) (Younus et al., 2014) (Supplementary Table 3). This massive expansion of GSTs in insects is possibly for meeting the requirements of metabolizing odorant molecules and resisting the damages of insecticides and/or plant secondary compounds (Durand et al., 2018). Based on sequence analysis, these 17 PiGSTs were classed into eight subcategories: three delta, two epsilon, four omega, two sigma, one theta, one zeta, three microsomal, and one unclassified (Figure 1).

**Figure 6:**
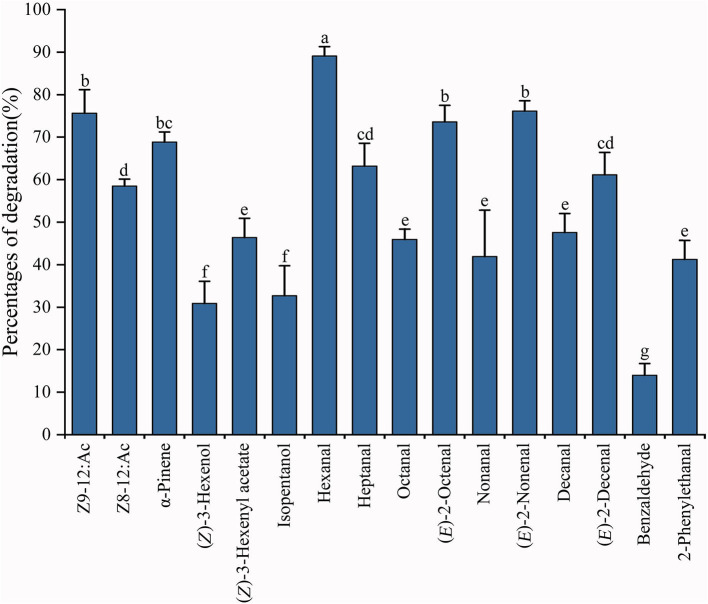
Degradation percentages against various substrates using recombinant PiGSTd1. Columns with different lowercase letters indicate significant differences at the 0.05 level by Tukey's HSD multiple range test.

Insect GSTs play various roles in degrading endogenous and exogenous compounds (Huang et al., 2017; Song et al., 2020). GSTs that metabolize specific substrates are usually expressed specifically in corresponding organs or tissues. For example, GSTs that function as pesticide-degrading enzymes are usually distributed in the insect digestive system, especially the midgut (Xu et al., 2015; Yang et al., 2020). Consequently, odorant-degrading GSTs are presumably antennae-specific. Tissue expression analysis indicated that the majority of PiGSTs genes were highly expressed in the abdomen of both female and male *P*. *interpunctella* with one exception that PiGSTd1 from a delta subclass showed significant antennae specificity (Figure 3). Multiple alignments of amino acid sequences revealed that PiGSTd1 contains conserved residues across antennae-specific GSTs with moths (Supplementary Figure 3). PiGSTd1 shares 65.31% identity with GST-msolf1 from *M. sexta*, which is involved in the degradation of aldehyde odorants (Rogers et al., 1999). Our degradation assays also verified that PiGSTd1 is a putative aldehyde scavenger in the odorant recognition pathway. PiGSTd1 showed low similarity (40.16%) to GmolGSTd1 (Supplementary Figure 3), which could efficiently degrade sex pheromone component (*Z*)-8-dodecenyl alcohol in antennae of *Grapholita molesta* (75.01%) (Li et al., 2018), suggesting different functions between two GSTs. The results of degradation evaluations are essentially in line with the sequence alignment: PiGSTd1 showed higher degradation efficiency to aldehyde compounds but rather lower efficiency to (*Z*)-3-hexenol (Figure 6).

Both FPKM and qRT-PCR results showed that PiGSTd1 expression was significantly higher in male antennae than in female antennae (Figures 2, 3), suggesting that PiGSTd1 is associated with recognizing sex pheromones produced and released from females (Kuwahara et al., 1971). The function of insect GSTds in degrading pheromone has been studied and verified in some moths. For instance, CpomGSTd2 is solely expressed in the antennae of *C. pomonella*, suggesting it is involved in odorant degradation (Huang et al., 2017). Our degradation evaluation showed that purified PiGSTd1 degraded 75.63 ± 5.52% of Z9-12:Ac, the sex pheromone component, in 1-h incubation. However, PiGSTd1 displayed lower degradation activities to Z8-12:Ac (58.47 ± 1.64%), a sex pheromone analog with a differently positioned double bond (Figure 6).

Besides degrading sex pheromones, insect delta GSTs also play roles in degrading host volatiles and environmental odorants (Li et al., 2018; Wang et al., 2021a,b). To evaluate the degradation activity of PiGSTd1 to host volatiles, we selected various volatiles from wheat flour or grains as substrates, including alkanals, 2*E*-alkenals, isopentanol, and phenylacetaldehyde (Uechi et al., 2007; Buda et al., 2016), as well as their analogs. Among all tested volatiles, recombinant PiGSTd1 showed best degradation activities to hexanal (89.10 ± 2.21%), (*E*)-2-octenal (73.58 ± 3.92%), and (*E*)-2-nonenal (75.81 ± 1.90%), which could attract *P*. *interpunctella* (Uechi et al., 2007; Buda et al., 2016). Unexpectedly, PiGSTd1 showed lower efficiency in degrading common green leaf volatile (*Z*-3-hexenol) and flower fragrance (phenylacetaldehyde), with degradation rates of 30.91 ± 5.17% and 13.97 ± 2.76%, respectively. Presumably, *P*. *interpunctella* infests processed foods and inhabits indoor areas, resulting in low degradation against green leaf volatiles and flower fragrances. However, how PiGSTd1 affects the olfactory recognition of *P*. *interpunctella* remains to be investigated *in vivo*.

In conclusion, we identified 17 PiGSTs based on antennal transcriptomic analysis of *P. interpunctella*, analyzed their phylogenetic relationships with GSTs from other moths, and investigated their tissue expression patterns. Furthermore, we cloned and purified the antennae-enriched PiGSTd1 and evaluated its enzymatic properties. The recombinant PiGSTd1 displayed GST activity to CDNB and high degradation efficiency toward pheromones and host volatiles. Thus, our results indicate that PiGSTd1 functions as an odorant degradation enzyme to ensure the sensitivity of the odorant detection system.

## Data Availability Statement

The datasets presented in this study can be found in online repositories. The names of the repository/repositories and accession number(s) can be found in the article/Supplementary Material.

## Author Contributions

The research was designed by JY and CL. The experiments were performed by HL, YT and HS. Data were analyzed by YT and QW. HL and YT wrote the manuscript. All authors have read and agreed to the published version of the manuscript.

## Conflict of Interest

The authors declare that the research was conducted in the absence of any commercial or financial relationships that could be construed as a potential conflict of interest.

## Publisher's Note

All claims expressed in this article are solely those of the authors and do not necessarily represent those of their affiliated organizations, or those of the publisher, the editors and the reviewers. Any product that may be evaluated in this article, or claim that may be made by its manufacturer, is not guaranteed or endorsed by the publisher.
