# Supplementary material for: Identification and Characterization of an Antennae-Specific Glutathione S-Transferase From the Indian Meal Moth
Source: Front Physiol. 2021 Aug 26;12:727619. doi: 10.3389/fphys.2021.727619 (PMC8427598; doi:10.3389/fphys.2021.727619)
Supplement: Supplementary file 1 [file Data_Sheet_1.docx]

Supplementary Material


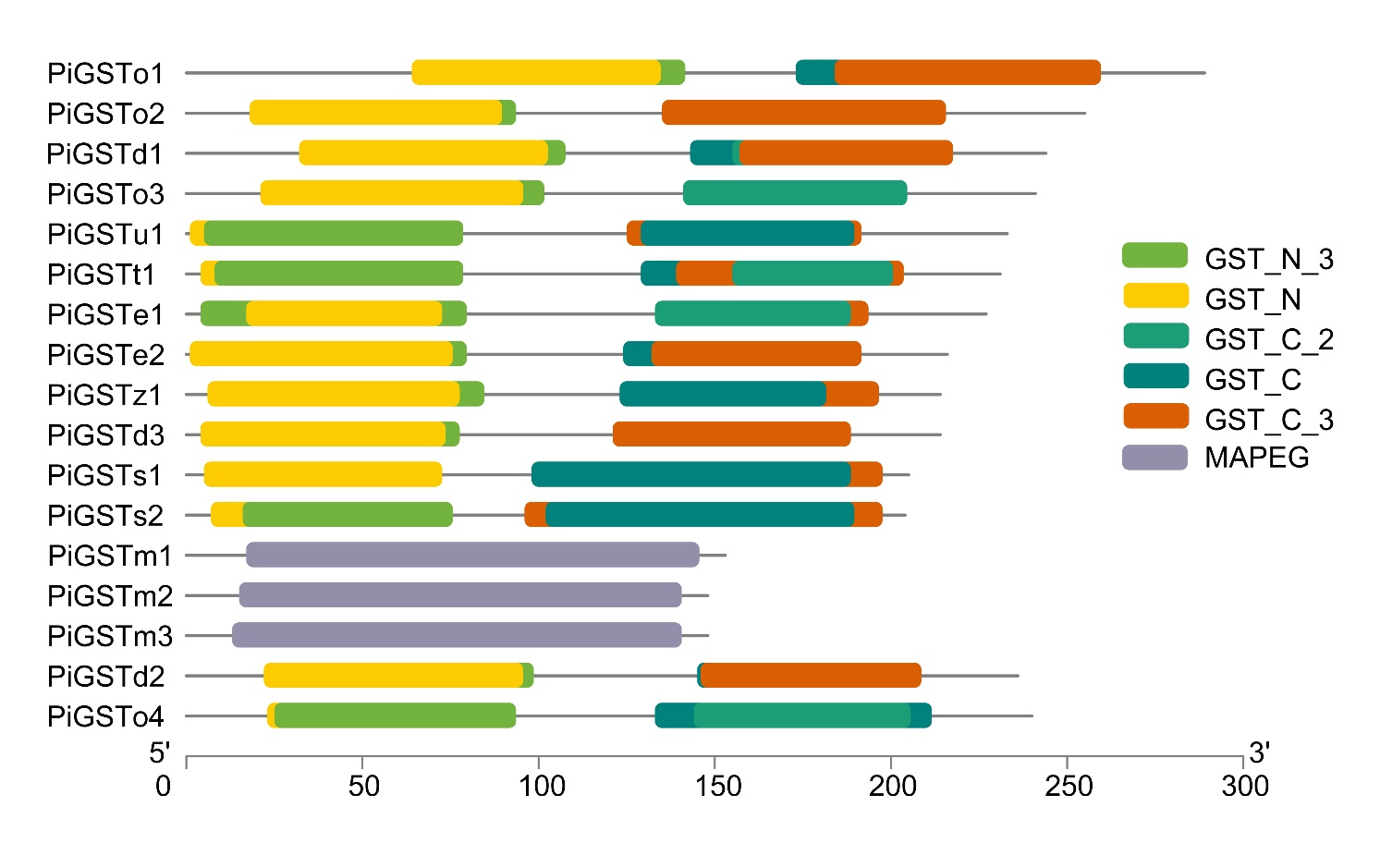


**Supplementary Figure S1** Conserved domains of PiGSTs


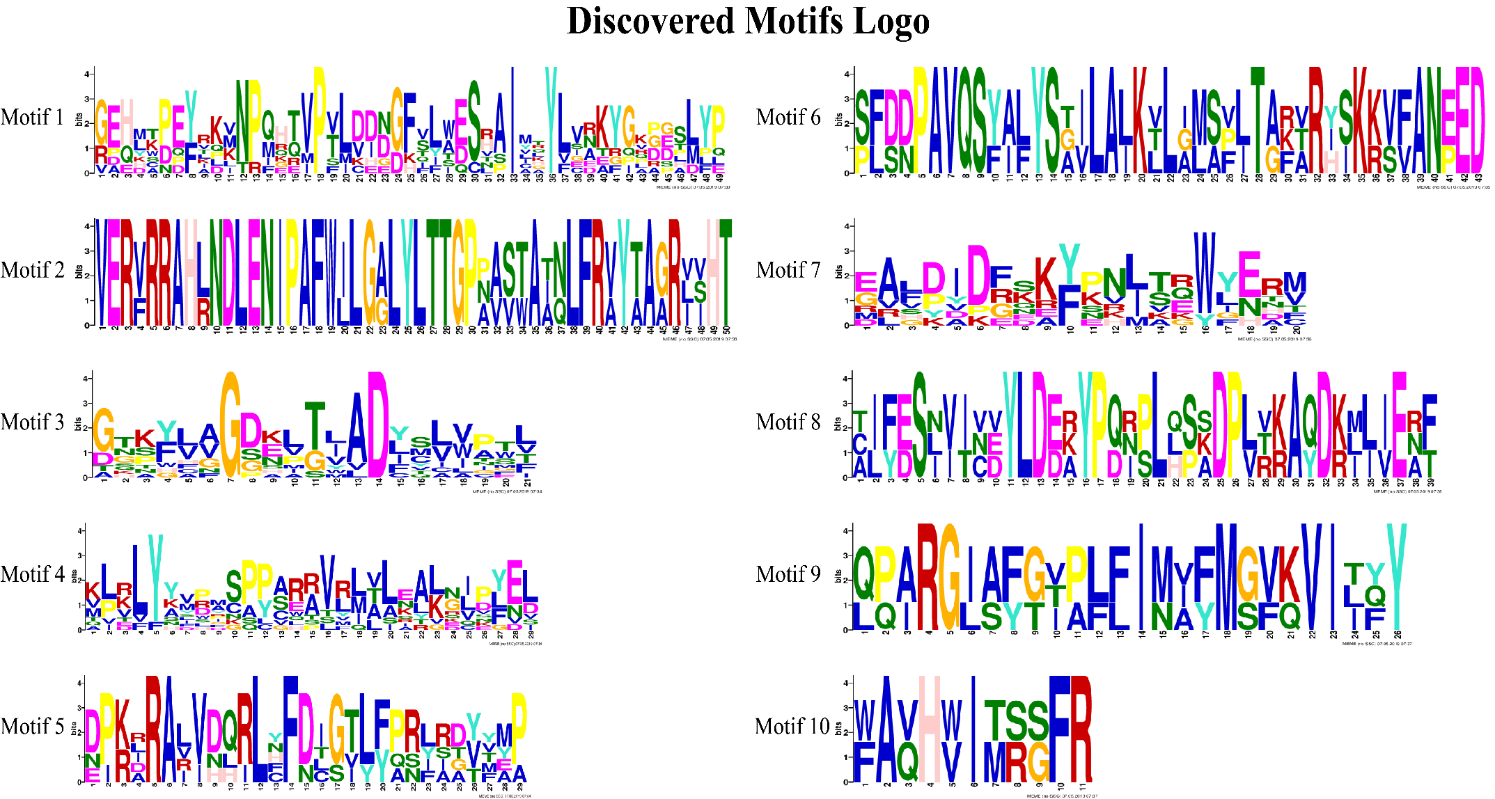


**Supplementary Figure S2** Sequence alignment of the conserved motifs logo of PiGSTs


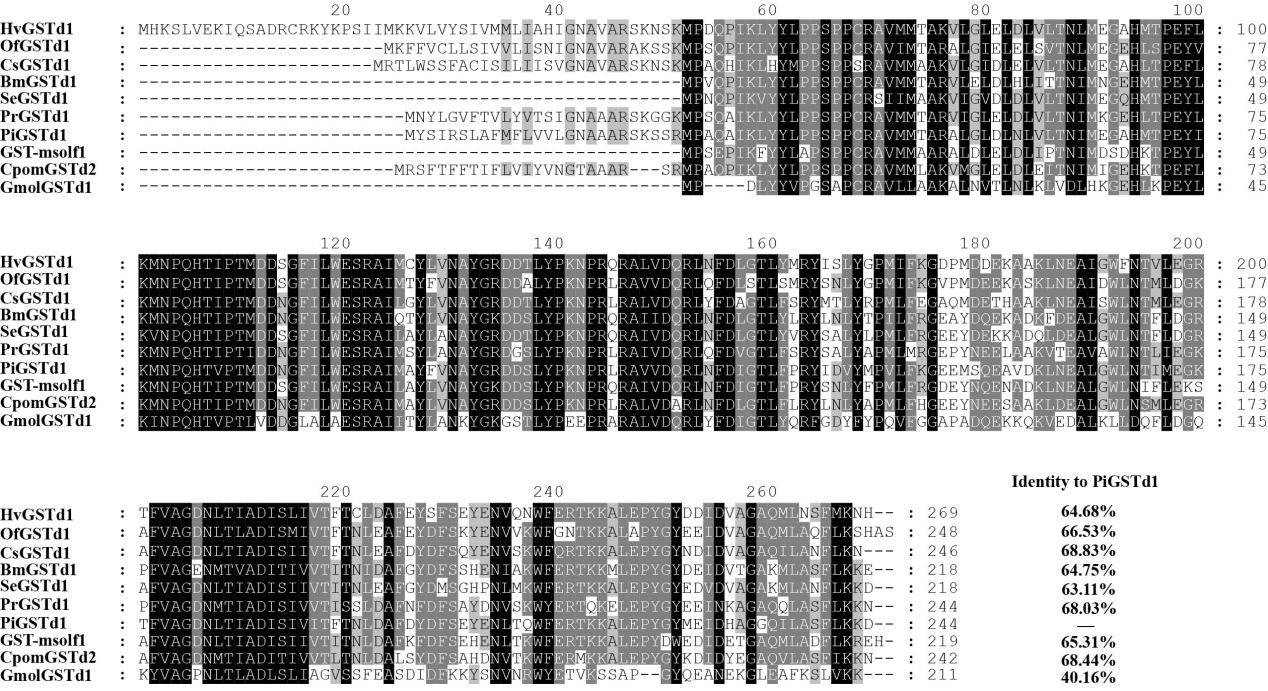


**Supplementary Figure S3** Multiple alignment of PiGSTd1 with delta GSTs from other moths, including HvGSTd1 (AWX68884.1), OfGSTd1 (QIC35737.1), CsGSTd1 (AKS40338.1), SeGSTd1 (ASN63930.1), BmGSTd1 (NP_001037183.1) and PrGSTd1 (APW77568.1), as well as three antennae-specific delta GSTs: GmolGSTD1 (MG696891), GST-msolf1 (AF133268) and CpomGSTd2 (KX500029)

**Supplementary Table S1**. Primers used in RNA interference and qRT-PCR.

| **Primers** | **Sequence (5'-3')**  **Forward** | **Sequence (5'-3')**  **Reverse** |
| --- | --- | --- |
| *β-actin* | GTATCAACGGATTTGGTCG | CACCTTCCAAGTGAGCAGAT |
| PiGSTo1 | CACCTTGCTTGCTCTCATC | CATGAATAGGACCCGTAGC |
| PiGSTo2 | GCCGACTTATCCTGGTAAAC | CAGACTGAGCAGAAGCAAAG |
| PiGSTd1 | GCTCAAGCCATCAAACTG | CGTTCACGAAGTAAGCCA |
| PiGSTo3 | CCAGCATAAATCATCGGAGG | GTTCTGCGGGTACCTCTCGT |
| PiGSTu1 | CTGGATGACGAGGGATTC | TGATGAGTTGGAAGTCCG |
| PiGSTt1 | CCATCTACAAATACCTGTCTCG | GGTCTGCTCTGGAGTGTTCT |
| PiGSTe1 | CCTTTACCCTGACGACATC | CTCAAGCATCTGATAGGCG |
| PiGSTe2 | GGCGTGGATTATGTCAATG | GAAACTCTCTGTGAAGCCG |
| PiGSTz1 | GCAATGAGTATCGGGATGTC | CACTCCTTCTTCTTCTCCTCG |
| PiGSTd3 | GCTCTCAACCTCAACCTGAAC | TAGCAGCCTTCTCCTTGTCG |
| PiGSTs1 | ATGGTGGGCATAACTTCG | CTTCTCAAACCGTGACAGG |
| PiGSTs2 | GCTGCTGCTGTCTTATGG | CGTCTTCCTCGTATTGGACT |
| PiGSTm1 | CCTGATGTTGAAAGAGTAAGGC | AATGGTATGCCGTATGCG |
| PiGSTm2 | GAGTGAAGACTGACGATAGGG | GCAGAACGGGATAGATAGC |
| PiGSTm3 | CAAGAGCTCGGTACAGCAAG | GAGGCATCGGCTTTATTG |

**Supplementary Table S2**. Substrate compounds used for degradation evaluation of PiGSTd1

| **No.** | **Compounds** | **CAS No.** | **Source** | **Purity(%)** | **Clarification** |
| --- | --- | --- | --- | --- | --- |
| 1 | 9Z-Dodecenyl acetate  (Z9-12:Ac) | 16974-11-1 | J&K | 93 | Sex pheromone |
| 2 | 8Z- Dodecenyl acetate  (Z8-12:Ac) | 28079-04-1 | J&K | 93 | Sex pheromone analogue |
| 3 | (+)-α-Pinene | 7785-70-8 | J&K | 98 | Green leaf volatile |
| 4 | (*Z*)-3-Hexenol | 928-96-1 | aladdin | 98 | Green leaf volatile |
| 5 | (*Z*)-3-Hexenyl acetate | 3681-71-8 | aladdin | 98 | Green leaf volatile |
| 6 | Isopentanol | 123-51-3 | aladdin | >99 | Grain volatile |
| 7 | Hexanal | 66-25-1 | aladdin | ≥99 | Grain volatile |
| 8 | Heptanal | 111-71-7 | aladdin | ≥99 | Grain volatile |
| 9 | Octanal | 124-13-0 | aladdin | 99 | Grain volatile |
| 10 | (*E*)-2-Octenal | 2548-87-0 | aladdin | ≥95 | Grain volatile |
| 11 | Nonanal | 124-19-6 | aladdin | 96 | Grain volatile |
| 12 | (*E*)-2-Nonenal | 18829-56-6 | J&K | >95 | Grain volatile |
| 13 | Decanal | 112-31-2 | aladdin | 97 | Grain volatile |
| 14 | (*E*)-2-Decenal | 3913-81-3 | aladdin | 95 | Grain volatile |
| 15 | Benzaldehyde | 100-52-7 | aladdin | ≥99 | Floral volatile |
| 16 | 2-Phenylethanal | 122-78-1 | aladdin | 95 | Floral volatile |

**Supplementary Table S3** Comparison of antennal GST repertoire of various insect species

| **Insect species** | **Delta** | **Epsilon** | **Omega** | **Sigma** | **Theta** | **Zeta** | **Unclassified** | **Microsomal** | **Total** | **References** |
| --- | --- | --- | --- | --- | --- | --- | --- | --- | --- | --- |
| *Spodoptera littoralis* | 3 | 15 | 3 | 4 | 1 | 2 | 2 | 3 | **33** | Legeai et al., 2011 |
| Chilo suppressalis | 4 | 3 | 4 | 2 | 1 | 1 | 1 | 0 | **16** | Liu et al., 2015 |
| *Cydia pomonella* | 1 | 1 | 2 | 1 | 1 | 1 | 1 | 1 | **10** | Huang et al., 2017 |
| *Epiphyas postvittana* | 3 | 5 | 3 | 6 | 1 | 0 | 2 | 0 | **20** | Jordan et al., 2008 |
| *Drosophila melanogaster* | 8 | 12 | 3 | 1 | 4 | 2 | 1 | 0 | **31** | Younus et al., 2014 |
| *Plodia interpunctella* | 3 | 2 | 4 | 2 | 1 | 1 | 1 | 3 | **17** | Present study |

**Supplementary Table S4** Sequences used in the phylogenetic tree

>PiGSTd1

MYSIRSLAFMFLVVLGNAAARSKSSRMPAQAIKLYYLPPSPPCRAVMMTARALGLDLNLVLTNIMEGAHMTPEYIKMNPQHTVPTMDDNGFILWESRAIMAYFVNAYGRDDSLYPKNPRLRAVVDQRLNFDIGTLFPRYIDVYMPVLFKGEEMSQEAVDKLNEALGWLNTIMEGKTFVAGDNLTIADISIVITFTNLDAFDYDFSEYENLTQWFERTKKALEPYGYMEIDHAGGQILASFLKKD

>PiGSTd2

YTPVASHFNFFINVKTQTDRMPIDFYYTPGSPPCRSVLLTAKALGLELNLKTLDLHHGEHMKPEFIKLNPQHCVPTLVDGDLVLWESRAIIVYLAQAYGKDDSLLPKDPKKQALVNQRLQFDVSTLYPAFSDQYYPWIFAGVPKSDDKEKKIHDALGFLDIFLGSSTWAAGDSVTVADLALVASISTIEAVGVDLSKYANVSKWFEKCKTTLVGYQEFNQKGIDGFKIMVANLTKK

>PiGSTd3

MPDLYYVPGSAPCRAVLLTAKALNLNLNLKLVDLHHGEHLKPEYLKLNPQHTVPTLVDDGFSIWESRAIITYLVNKYGKGSTLYPEEPKARALVDQRLYFDIGTLYQRFADYFYPQVFGGAPADKEKAAKIEESLKLLDQFLEGQKYVAGPNLTVADLSLIAGVSSFEASDIDFKKYNNIKRWYETVRATAPGYQEANEKGLEAFKALVNSMKK

>PiGSTe1

MPVLYKSDASPPARTVLMVSELLGVVLELRELNPVLREQDAPPYRQKNPMRTVPVLDEGDFSLADSHAIVLYLIAKYGKPEHMYLYPDDIRIRARIHLILFFDCGVLFPRLRTVMAPTYGGKLGELSKRMIHNIDDAYQMLEEYLNDGSYLAGDAMTIADLSVLTTTSSLHGLHPVDEKRFPKLTRWLENMNQKEVCQRINAPGSQLHVEGLKALMAHHRENQKSKL

>PiGSTe2

MVLTLYKIDASPPVRAVFMTIEALNIPGVDYVNVNLLEREHLKDDFVKINPQHTVPTLKDDDFVLWDSHAIAGYLVAKYGADDSLYPNDPKRRALVDQRLHFDTGILFPSLRGTVEPIFFWGEKSPRPENLEKIEKAYGFTESFLTSPWLVGGEVTLADICCVATISSMNEVLPIDGNKYPNLVGWLERCEKEIFYKKGNESGLLQFRQLLKSKLQ

>PiGSTo1

MITSTLTFAVHVIMSSFRVLAPVARLLENAFPDRNMASKAVKGKVNFNSKHLRRGDPMPPYTGKLRVYNMRYCPYAQRTLLALIAKDIDFEVVNIHLMDKPEWFLARSAFGKVPALEVADGVTIYESLVTVEYLDDAYPQRPLLPKDPVTKAYDKIIIEATGPIHGLFYRILRTPESVTNEHVEAYHKALKFIEDQIKARGNKFLGGSKPGYADYMVWPWFERIINSDLDGRLKIDGQKFKVLSEYINHMLQDPVVSQYIVPKEVMSQFIIPYRSGDFANINYDIMIEE

>PiGSTo2

MSEKHLQTGDALPTYPGKLRLFAMRFCPYAERSVLVLNAKKLSYELVFINLDHKPEWIFKYSPKGTVPALEYEEGKAIFDSNIINVYLDEKYPDISLQSADPLRRAQDKMLVENFASAQSAYYTAAFNSQALQPSNVENYHKGLELLQKEIETRGTKFLNGDEPGLVDYTLWPFLERFEALPLIGKSEFAFDKDKYAALIAYMNVMKSAPAVQAYRLAPETHAKFTESRAKGDPDYNMLDSSDTVCCMRPRKKKE

>PiGSTo3

MSYYQHKSSEAPPPPPLPPGQLRLYHVHMNPYAHRVRLVLEAKRVPYELHALDPLRLPDWFREKNPRLKIPVLEVPTPQGDRCLFESIVICDYLDERYPQNPLHSSDPLVKAQDRLLIERFNELIKGSLECFDTNFTFGGDQILQTVDVFEKELAARGTPYFGGSSAGMLDYMVWPWLERLCLLRCLRPRAFDAKRDAFPHMSQWGHAMQCEAVVKQLASSPERYLQYYRSARAHAMGYCL

>PiGSTo4

NILPVIKVIENLSVAMATNQEHVKLLGATGSPFVCRVQIALKLKGIEYEFVEENLATKSEQLLKYNPVHKKVPVFVHNEKPISESLVILEYIDEVWKQNPILPSDPHQRALARFWSKFIDDKIVSTSFKSVFSLDEKEREKNIEEATEALHFLENELKDKYFGGEEFNFVDIAAVFVAFWVPLVQDITELQLFTAEKFPKLYNWSQEFLDHPVVKETLPPREPLFAFFKGRYESLLAASK

>PiGSTs1

MSKKLLYFSIGGIAEYIRYMLHYGGHNFEDVRFDYKKWPIKEVKDSLPYGQLPLYEEGDRKLNQSIAISRYIASQTGLLPSDPWEQAILDAAVLNVNDFRLKSYSYFIEKDETKKEQIKKEFLTDSVDFYLSRFEKELKDNNGHFGGKLSWADFYLVGMLEGINLAMNVQLEKNYPSITALMKEIPSLPGVKEYVAKRGPYVFPA

>PiGSTs2

MSKVEFYYFPIKALGEPVRLLLSYGGQEFEDNRVVPEKWPEFKPKTPFGQMPFLVINGKQYAQCTPICRYLGRKYGLAGADLEEDFEIDQNVELVHDLRAKAAQVQYEEDAATKAKRHEKYTREIYPNLLTKLDEIIRKNNGHLALGKLTWGDFIFAGIYEYLKVMLQAPDLDEKFPSLRALEQAVLNLPKVKEYVAKAPKCDY

>PiGSTt1

MGKMTLKLYYDLMSQPSRALYILLKTVNCKFESKYVDLRKGEHYSDEFTKVNRIQRVPVIDHNGFVLTESVAIYKYLSREGIVPESLFPKESQQQARVEEFLEWHHIGLRLHTAMYFRAVYLDPIIFNRKNTPEQTVGYQRRMETCLEDFSTKWLGRGHAFVVGDKVTVADLVAACELEQPRMAGYDPREKFSNIAEWYERVRSYFNPHYDEAHVIVNKVIAKNNKTNAKL

>PiGSTz1

MAKPVLYSYWRSSCSWRVRIALNLKEIPYDIKAVSLIKGGGEQHCNEYRDVNPMEQVPSLCIDGHTFIESLSIMHYLEETRPQRPLMPQDCFKRAKVREICEVISSSIQPLQNLIVLIYVGEEKKKEWAQHWITRGFRAVEKLLSASAGKYCVGDEITLADCCLVPQVFNARRFHVDLRPFPIILRIDRELEQHPAFRAAHPSAQPDCPPEVAK

>PiGSTu1

MVLKLYAVSDGPPSLSVRQALARLQIPYELIDVNFNAGDHMTPQYALMNPQKEIPVLDDEGFFLSESNAILQYVCDKFQPGGPLYPADPKDRAIVNHRLCFNLSTYYANISAYTMAPIFFDYERTPFGLKRVNMALDVFETYMERLGTSHAAGDHLTIADFQLINSTMTLEAVDIDFSKYKRITKWYNDFKSNYPDLWKISADAMKIIQHFAANPPDLSHLNHPIHKARKTNK

>PiGSTm1

MSSFIFSFDDPAVQSYAFYSGVLALKTLGMSVLTAKVRHSKKVVANEEDAKLDKKAVIKFDDPDVERVRRAHLNDLENIPAFWLLGGLYLTTGPPASTAINLFRVYTAGRIVHTLVYAIKPLPQPARGIAYGIPFFIMVFMGVKVIIYYSKSL

>PiGSTm2

MAAIPFDNPAVQSFILYSAILAIKLLILSPITGFTRIIKRSFANPEDSKFLGGRVKTDDRVERFRRAHLNDLENIPAFWILGALYLTTGPNVVWATQLFRVYAAARVSHTIFHAIYPVLQIRGLSFTTALLINAYMSVQVILQYITAL

>PiGSTm3

MVSLSDPAVQSYALYSTILALKVLAMAFLTARARYSKKVFANEEDAVATKGKVKYDDPDVERVRRAHRNDLENIPAFWILGALYLTTGPAASTAANLFRAYTAGRLIHTFVYAIKPMPQPARGIAFGVPLFIMIFMGFKVITFYASAL

>PxGSTd1

MPVEPIKLYYFPPSPPCRAVMMAAKAMGIELEMVLTNIIEGEHMTPEFLKMNPQHTIPTIDDNGFILWESRAIIQYLANAYGRDDSLYPKNPRLRAMGDQRLNFDLGTLVSRYLNLYSPVLHGEPFSDDMDAKLKEALGWFNTMLEGRAFSAGDNLTVADISIVVVFSNLEAFGYDFTAYDNVSKWFERTKKALEPHGYKEIDQAGAQMLASFIKKD

>PxGSTd2

MPDLYYVPGSAPCRAVLLTAKALNLNLNLKLVDLHHGEQMKPEYLKLNPQHTVPTLVDDGLAIAESRAILTYLVNKYGKGSTLYPEDPKARAIVDQRLYFDIGTLYQRFGDYFYPQLFGGAPEDKEKLAKVDEALKFLDTFLEGQKYVAGNNLTVADLSLVASVSSFEAANIDFLKYGNVKRWYETVKATAPGYEEANGKGLEAFKGLVNSIMKK

>PxGSTd3

MAIDLYLTPGSAPCRLVLLTAAALNIQLNLNHVDLRAGEQFSPDFLKLNPQHTIPTIVDQGFALWESRAISRYLVNKYGHGSTLYPEDPQSRALVDQRLDFDLGTLYPKFADYFYPQVFGSAPADPEKLKKLHEVLGFLDIFLGDEKYAAGSDLTIADLSLVATVSTIDAAGISLDDFPNIHRWFELVKSTAPDYENANGKGIKAFKELVAQLNAKTEL

>PxGSTd4

MPAIDLYEMPSSAPCRAVKLTARALGVPVKLHLVDLMAGEHLKPEFTKINPQHTIPTIVDDGFTLWESRTIMRYLVNKYGKGSSLYPEEPKARALVDQRLDFDLGTLYDRYAVYFYPQIFGTAPENPELLKKLHEALAHLNHFLGESKYAAGPNLTIADLTLVVTVSTIDLWEIIDFKQYPNIDKWYEHLKSSVEGYEEENLAGLGKFRAFIKEFKAKKAAAK

>PxGSTd5

MPAIELYEMQGSAPCRAVRLTARALGKPLTVHHLDLMAGEHLKPEFVKINPQHTIPTIVDDGFALWESRTIMRYLVNKYGKGSSLYPEEPKARALVDQRLDFDLGTLYAKYAVYFYPQVFGTAPENAEDLKKLNEALAHLNTFLGESKYAAGSNLTIADFSLVATISTIDVSDIVDLKQYPNIVKWYEHLQSSVEGYEEENLAGLENFRSFIKEFKAKKAAAK

>PxGSTe1

MMRKLLFRNSPTDLVRCKSITLYGDEISPPVRFVQMTADLLGIQYKFKKVDLFKQENKQDFFKKINPLQKVPALKVGDTVITDSHAIAMFLCENSDGQTLYPDDPIIRPIVQQMMFFNSSTLFNIDSTIYSNFFAGSESIDANLVKDWTLALDYLEYQLRKHEWLAYDKMSLCDLCCGATVSTLQLLIPPTEKHKKVNRWIKRLEEIPCHSINKIGLDRLQFLIDQIKDVAH

>PxGSTe2

MSKLLLYKVDGSPPSNSILMIAHLLNLDMDYKEPDLLRLEHRSPEFKKINPMGTIPVLKDGNFVLAESHSILKYIVEKYGGPPRSTLYPCARRPRAAVDERMHFDTGVLFISLSSVVKASIFGDQPAVTPEQLASIESSYATLELYLERSRFVAADHLTIADFGVLSTTLALRHILPIDANKFPKISAWLSQLEEEAFVKNVGAPNMEKFKAILYSSWERNKSKMAR

>PxGSTe3

MPLILYVSDASPPARAVLMLAEILQLEFDKQYINPVLREQDSPEMTQKNPMRTVPTLEDGEFCLADSHAIILYLMEKYGQNHRNLYPEDLRVRSTIHQRLFFDCSILFPRLRAIMAPTFLGKLSQPSGSMVTNIEDAYRTLEAYLSQSKYLAGPEMTIADISAVATVSGLDGLHPIDEKRYPNTREWLVNMSRKTFYRKCNAQGNEMLISLLKSSMENNKQNDKKAKL

>PxGSTe4

MGLTVYKIDWSPPARAVIMTLEALNITDAELVDVSLLDGKHMSEEYLKMNPQHTVPVIKDGDFVLWDSHAICAYLVDKYGKDDSLYPKDLQKRAVVDQRLHFDTGILFPSVRGAAEPVLFDWEPTFNPEKLKVIQSGYDFLEKFLDHSYLAGDHLTIADICAGATVSSMNVIVPIAANRYPKISAWLDRLNSIEYFSRINGNGIKIITALFESKLNKSKK

>PxGSTe5

MVLTLYKLDASPPARAVMMTLEALGIRDVEMVDVNLFEGAQFTEEYVKMNPQHTIPALKDDDFAIWDSHAICPYLVSKYGQDDSLYPQDPQRRAVIDQRLHFDSGILFPSLRATVAPVLFLGERSFKPEGLQAIKAGYDFLEKFLDKPYCAGDQLSIADICTSATVSSMSAALPIDADTYPNITAWLDRLSKEEFYTKVNLPGLQQFSGALKSKLL

>PxGSTo1

MSEKHLQTGDALPPFGGKLRLFAMRFCPYAERSVLVLNAKNIPYDLVFINLDQKPEWIFNFSPRGAVPALEYEQGKGIFDSNVINVYLDEKYPEVPLQAADPLRRAQDKLIVENFSAAQSAYYTAAFNAQALQPSHLENYHKGLELLQKELETRGTKFLHGDQAGLVDYTLWPFLERFEALPLLGKSEYAIDKSKYDILLTYMESMKQVPAVKTYYLSADMHAKFTESRVKGDPNYNMLDSSAEVCCFRPRKKKE

>PxGSTo2

MSTRGIKFNTKHLRKGDPLPPYNGKLRLYNMRYCPFAQRTVLALNAKDIDYEVVNINLFEKPEWLTSKSAFGKVPSLEIKEGLSIYESLVTVEYLDEVYPQRPLLPKDPVQRALDKIIVEACTPIQGLFIKLIKFPESISEDTVAAYHKALHFLQEQLQSRGTRFFGGDQPGFVDYMIWPWFERVLPYQKVESRVQIDAGKFKLLLEYLQNLKQDPVVKQYLIEDEVLFKFLEPYKTGGEPNYDLLLEA

>PxGSTo3

MSFYYQERPAGPTPPGPLSNKLRLYHVDMNPYGHRVLLILDAKKVPYEVCKLDPLRLPEWFREKNPRLKIPVLEIPTDQGDKYLFESIVICDYLDERYTRNPLHSRDPFVKAQDRLLIERFNELIKGSLECFDTNFAFGNEQIIQTVNIFEKELESRGTVYFGGDRPGMLDYMIWPWIERLYMLRCLNPTKFDEKRHIFPNFADWGDQMQLDEVVKKHASSPEDNFEYYKNARAHSMGYYL

>PxGSTo4

MSTRGIKFNTKHLRKGDPLPPYNGKLRLYNMRYCPFAQRTVLALNAKNIDYEVININLFDKPAWLTSKSAFGKVPSLEIKEGLSIYESLVTVEYIDEVYPQRPLLSKDPVQRALDKIIVEACTPIQNLYIKLIRFPKTFTEDTVTAYNKALEFLQEQLKIRGTKFFGGAEPGFVDYMIWPWFEQVLSYQKIDSRAKIDAEKFKTLLDYLQHLKQDPVVKQYLIEEDVLFKFLEPYKTGGEPNYDLMLDA

>PxGSTo5

MTEKHLQTGDALPPYNGRLRLFAMRFCPYAGRCVLVLNAKNIPYDVVYINLTKKPEWIFNFSPKGAVPALEYEQGKALFDSNVINTYLDEEYPEVPLQASDPLRKAEDQLVVENFNTVITAYFTAAYNPQGLQPSHPEIFHRGLEQLQKELETRGTEFLHGEEVGMVDYTLWPFLERFNTLPLGNAEFALDKSNYETLLTYMESMKQLPAVMSNYLPPDIYAKFTDSFVTGDPDYNMLLSNDEGC

>PxGSTs1

MAKKLHYFDVNGIGESIRYILHYGGQKFEDVRYQISGWPDKKVKDALPFGQLPLYEEGDRSLHQSLAIARYVASQSKLLPNDLWEQAVVDSYVMTIYDFWFTKVIPFVKETDAAKKATLKKEILDESIHFFFSRFDKVLKEHNGFFIGKLTWAEFILVGIIEAGNLFLDYEIEKNYPHVKAAVQKVLTLPGVKEYIAKRKVYAL

>PxGSTs2

MPVVKFYYFPIKALGEGPRLLLAYGGQEFQDIRVDKESWPEFKPKTKYGQMPILEIDGKQYAQSAAICRYLASRYGLTGADAEQNFEIDEAVDFFNDIRAKAAQVHYEEDEKVKEKRHETYSQTVYPDLLGKLHDIVQRNNGHLAANKLTWADFYFAGVYDYMKVMLRRPDLDQQYPGFAKVYETVYSLPKVKAFADAAPKTDF

>PxGSTt1

MSQPSRAVYILLKKSNINFEPKYVDLRKGVHYTDEYSNNINRFKKVPVIDHNGFILTESVAIIRYLGRENVLPEALFPRADKVLNTRLDEFLEWQHLGLRAPLAMYFRVVMFSPDSEKIPSYQKRMETALDEFSTLWLGRGNQYILGDTATVADLLAACEVEQPRMTGYDCTANYPVIREWMDRVRSYFNPHYAEASSIVEKIAAKRIPMKKPTAKL

>PxGSTu1

MVLKLYAVSDGPPSLSVRQTLAALQLPHELVSVNYGAGEHLTEGYAQMNPQKEIPVLDDDGFFLSESNAIMQYLCDKYKRDSPLYPTEAKARAIINQRMCFNLASYYANISAYTLAPIFFDYERSSLGLKKVHLVLEVFDTYLARLGARHAAADHLTIADFPLVNSTMTLEAINVDFSKYERVRKWYNDFKRNYPDLWKISEDAMKELKQFSAHPPDLSNLNHPFHPIRH

>PxGSTz1

MAKPVLYSYWRSSCSWRVRIALNLKEIPYDIKAVSLIKGGGEQHCNEYREVNPMEQVPSLCIDGHTLIESLSILHYLEETRPQRPLMPQDCYKRAKVREICEVISSGIQPLQNLVVLIYVGEEKKKEWAAHWMTRGFRAVERLLSGSAGKYCVGDEITLADCCLVPQVFNARRFHVDLRPFPIILRIDRELEHHPAFRAAHPSTQPDCPPEAAK

>PxGSTz2

MASPAVLHGFFASSCTWRVRAALVLKSIPFEERHVDIVQLKTHLSDQYQAVHPAQKVPALEIDGTTLVESMAILQYLEDTRPRPALAPAAPLPRARMREIVETIVSGIQPLQNVGVRGLLGSDEEYSAFSRGAARRALQTLEALLARSAGQYCVGDQLSMADLCFVPQLFNAVGRLKLDISDLPTISKLYAKLSKEEIFMKTHPRTVKHLSET

>CpGSTd2

MRSFTFFTIFLVIYVNGTAAARSRMPAQPIKLYYLPPSPPCRAVMMLAKVMGLELDLEITNIMIGEHKTPEFLKMNPQHTIPTMDDNGFILWESRAIMAYLVNAYGRDDSLYPKNPRLRALVDARLNFDLGTLFLRYLNLYAPMLFHGEEYNEESAAKLDEALGWLNSMLEGRAFVAGDNMTIADITIVVTLTNLDALSYDFSAHDNVTKWFERMKKALEPYGYKDIDYEGAQVLASFIKKN

>CpGSTd3

MPIDLYYLPYSPPCRPVLVLADALKLKLNLKELNTRAGEHLTPEFKKINPQHCLPTLVDDDLTLWESRAILTYLASKYGDGSLYPADHKLRAAVDQRLYFDMGTLYQTFTDYLYPQLFGNQPADEEKLKKLEEALEFLNIFLEGKPWVAGSTLTVADYSIICTVNSIIALDVDVYKYPNIKEWYERAKSSMPGFNVSEQVIELLKKLFQDFKAKNTSV

>CpGSTd4

MDLYYTPGSAPCRLVLLVAAALDIQLNLKELNLQAGEHMTTEFLELNPQHTVPTLVDDGFALWESHAICRYLIRKYNNRELYPEDIQIRALIDQRLDFDLGTLYPRFRNFFYPQVFAGKPANESLFRNLKESLDIFNSFLKGHKYAVGDTLTLADLSLVATVSTLEAAGVSLESYPRVERWFELVKSTAPAYEEANEKGLIKFKALVANFKAKTEL

>CpGSTe1

MVLTLYKLDASPPVRAVKMVIAAINLPDVEYVDVNLLQGDHLKEDYIKINPQHTVPTLADDDFVIWDSHVIATYLINMYADNDSLYPSDPKTRALIDQRMHFDCSILFPPIREAVGPVIFGNDKAFQPEVLHKIQSGYEFTEKFLTGEWLAGDDLSVADICCVATISTSNEILPIDETLFPKLAEWMKRCSELEIYKNENEPGLNIFSQILKSKLA

>CpGSTe2

MVKLYKLDGSPPARACMIACELFNVPVELIDVNLMAGEHLTPEFLKKNLLHTVPTLEDGSLVIHDSHAALMYLADVYGKQESFYPKETKQRAHVNQKLFFNSTILFPRMRNTTYPIFMEGMTEIPQKNLDAIEEAYGFVEEFLSRTKYLAGDNITIADIAAYPTVTSLLIFLELNAQKYPKTQAWLKDLEKLPYVQKGNAKGLEDLSNFIKSKLG

>CpGSTe3

MRAILYHSNSSPPSRAVKMVAGILKVQLEERYLNPVARDQDTPELIEKNPMRSIPMLDEGDYWIADSHAIIVYLFEKYAKPEHQHLYPSDIRKRATINQRMYFECGILFPRLRSVMAPTFWGKLTELSKSMKSNIEDAYRTLEAYLSRNLYVADDVLTLADISIVTTVSTMDGIYSVDGKRYPKLKQWLQTMSQKDFCQKYNEPGREELVTVLVTMMDNNKHNQRAKL

>CpGSTz1

MAKPVLYSYWRSSCSWRVRIALNLKEIPYDIKAVSLIKGGGEQHCNEYREVNPMEQVPSLCIDGHTLVESLSIMHYLEETRPQRPLMPQDCFKRAKVREICEVISSGIQPLQNLIVLIYVGEEKKKEWAGHWITRGFRAVEKLLSASAGKYCVGDEITLADCCLVPQVFNARRFHVDLRPFPIILRIDRELENHPAFRAAHPSSQPDCPPEVAK

>CpGSTt1

MTLKLYFDLMSQPSRALYILLKNIKCNFTPVPVDLREAEHYSEEYTKINRFQRVPVIDHNGFVLTESVAILKYLSREGIIPDSLYPKDSKQAARVEEFLEWQHAGLRLHCAMFFRVKALDPIITGKTPDPKTLQGYERRMENALGTFNDLWLGQGKPFVAGDHISVADLLAACEVEQPRMAGYDAIAKYPNIAEWMGRVRDHFNPHYDEGHVILNKIVRNKSKMAPKL

>CpGSTs1

MPQTEFYYFPIKALGEPIRLLLAYGGEDWKDNRVTPEDWPNFKPKTPFGQMPVMVVDGKQYAQSIPLSRYLGRKHGLAGSNIDEDFLIDQNVEFVNDIRAKAAQVQYEPDEALKAKKHEDFSKNVYPGMLAKLDQILKENNGHLALGKLTWGDFVFAGIYAYLKVMLQAPDLDERFPAFKKLEQTVYSLPKVKAYADAAPKTDH

>CpGSTo1

MSYFQSRYAGSVPPPPLGDKLRLYHVDMNPYGHRVLLILEAKKTKYEVYKLDPLRLPDWFRTANPRLKIPVLEIPTDQGDKFLFESVVICDYLDEKYGRGQLHSRDPYVKAQDRLLIERFNELIKGSLECFDTNFAYGSEQIVQTLDIFEKELTARGTNYFGGDRPGMLDYMIWPWVERLYLLRCINERKFDEKRSLFPNFADWGDQMQLDEVVKKHANSPAEYFDYYKNARTHSMGYYL

>CpGSTo2

MHTLALASWRVLMPVVQNLKYVLPLYRAMAGTAKINFDTKHLKKGDPLPPYNGKLRIYNMRYCPYAQRTILVLNAKQIDYEVVNINLKEKPEWLTSKSLFGKVPAIEVADGVCIAESLVTSEYLDEVYPQRRLLPKDPLKKALAKIIVEGSGPIQTMLFKILRTPDQVTEENIAAYRKYLTFIQDELKKSGSKFLGGSEPGFADYMIWPWFERMVPISELYDAVKIDETEYKLLWEYITNMFKDPAVSQYVVPKEILFKFMEPYRTGGTSNYDLLTVE

>CpGSTu1

MVLKLYVVSDGPPSLSVRQALALLEIPFQQIDVDFCKGEHMTEEYALMNPQKEIPVMDDDGFFLSESNAILQYICDKYQPGTPLYPLDPQIRAVVNHRLCFNLSTYYANISAYTMAPIFFDYERTPLGLKKVHMALAVFETYLARAGTTHAAADHLTIADFQLINSTMTLEAIGLDFSKYEKVSQWYEDFKKDYPELWKISEDAMKEIQHFNANPPDLSHMNHPIHPIRKIKK

>CpGSTm1

MGVSLKDPAVQSYIVYSAILALKLIALSSLTGMKRITKKVFANPEDAAALKGKVNLNDPEIERARRAHLNDLENIPAFWLLGALYVTTGPVAAWATLLFRVYTVSRFIHTFVYAVVPMPQPARGIAFGIPYFIKWYMGIQVILHYAAAL

>CpGSTm2

MAISLQDAVVQSYIVYSAILALKLLAVSTMTSMKRISRKVFANPEDAVGLKGKVNLNDPEVERVRRAHLNDLENIPAFWLLGALYVTTSPVAAWATLLFRVYTVSRFIHTFVYAVVPLPQPARGIAFGIPYLIKWYMGIQVILHYATAL

>BmGSTd1

MPVQPIKLYYLPPSPPCRAVMMTARVLELDLHLITTNIMNGEHMTPEYLKMNPQHTIPTMDDNGFILWESRAIQTYLVNAYGKDDSLYPKNPRQRAIIDQRLNFDLGTLYLRYLNLYTPILFRGEAYDQEKADKFDEALGWLNTFLDGRPFVAGENMTVADITIVVTITNIDAFGYDFSSHENIAKWFERTKKMLEPYGYDEIDVTGAKMLASFLKKE

>BmGSTd2

MTIDLYYVPGSAPCRAVLLTAKALNLNLNLKLVDLHHGEQLKPEYLKLNPQHTVPTLVDDGLSIWESRAIITYLVNKYAKGSSLYPEDPKARALVDQRLYFDIGTLYQRFSDYFYPQVFAGAPADKAKNEKVQEALQLLDKFLEGQKYVAGPNLTVADLSLIASVSSLEASDIDFKKYANVKRWYETVKSTAPGYQEANEKGLEAFKGLVNSMLKK

>BmGSTd3

MAIDLYFTAGSAPCRVVLLVAAALDLQLNLKPLNLWEREQLQADFLKLNPQHTVPTIVDEGFPLWESRAISRYLVNKYGGDSSSLYPKDLMARALVDQRLDFDIGTLYPRFAQYFYPQVFGGAKPDAAALKKLEEALVFLNAFLEGQKYVTGDVLTIADLSLVATISTIDAAEISLKSYPNVEKWFELMKTTAPDYQNANQKGIDEFKKLIAQMKAKTEL

>BmGSTe1

MVLTLYKMDASPPVRAVYMVIEALSIPNVKYVDVDLLAEDHLKEEFLKLNPQHTIPMLTDDKFVIWDSHAIATYLLNKYGKGSSYYPEDPEKRALIEMRLHFDSGILYPALRENDEPIFFWGETTFKPEGLAKIKSAYDFTEKFLSDSPWIAGDDVTVADMSCVATIGSLDALLPINEKEYPKITSWLKRCSELDFYQRGNNVKGLLEFKALLKQYLSRGKE

>BmGSTe2

MSIIIYQTSVSPPARSALMVVNILGIKAETREVTLPRRDHYSQEYLEKNPLHTVPILEEDDLVIADSHAIITYLVSKYGEEKHESMYPKDLKIRAITDQMLYFDATILFPRLKTVIYSVVRGQGMSRQQIADIQEAYDVLEIYLSKNIFVAGNEFTVADISCVATLSSLDCVLPVDKKHVNVNRWWATLSNEKWYKEVNVPGLELFRSFIKQFLK

>BmGSTe3

MSLMLYKLNASPPARTAMMVCELFKVPVKMVDVNLSKGEHFSPEYLKRNPLHTVPTLEDGDLIITDSHAIAMYLADKYGKDDSLYPKDLKSRAIVNQRLFFDSTVLFSRMRSVTFPVIIEGCKTVTEKQINDIIEAYGYVETYLSNTKFIATNNLTIADISAYAVVSSLLFIVPLDGAKFPKTQTWLNEMEKKPFAQKYNVNGVAELGALLKEKLGS

>BmGSTe4

MVFILYKKDTSPPCRSVQMVLHELGIYDVELIEVNLPERDHLKEEFLRMNPQHTVPTLIDGDFIIWDSHAIVTYLVNRYAKNDTLYPKEPKQRAIVDQRLHFDTGVLFAILRATAEPVLYNNEKSFKQENLEKMEAAYEFVEKFLTSDWLAGDQVTLADICCVSTISSMNVIVPIDKKKYPKIISWLQRCSEQEFYKKANEPGLKKFIEMFKNKIGN

>BmGSTe5

MVLTLYKLDASPPVRSVYMVIEALKIRDVEYVDVNLLEGSHLKEEFLKMNPQHTIPLLKDDDFLIWDSHAISGYLISVYGADDSLYPNEPKKRALIDQRLHFDSGILFPALRGVAVIIFFNLLCLGQDELIIFRGEKEIRPENLAKIKSAYDFTEKILSSDWIAGDEFSLADICCVTSISTLNEMVPIDGSLYPKLASWLDRSSQLPIYKKANEPGLLQFREIFKNKTS

>BmGSTe6

MTPILYKTDASPPARAVMMIVDILGLKVDEQELNPILRQQDTPEFKKKNPMRTIPILEEGDFYLADSHAIMLYLIDKYGKPEHAHLYPSEKRKRATINQRLFFDCGVLFPRLRAVMAPTYAGKLAELNRNMIKNIEDAYSIMESYLTENLYLADEVVTVADISAITTISSLNGLYPVDEKSKWINRMNDKEYCRKINTPGSELHVAGLIALMDNTKHNQQSKL

>BmGSTe7

MMTAAHLNIQLDFHPIDLFKSEHKMDAFNQINPLQKVPVLKVDKDVICDSHAIAMYLCETTKSEDLYPGSALARAKINQMLFYNATTLFPIDSFILSEYFCGKMPSGVKVDEWFNSLHYLNTALSVSPWLAGDQMRICDFCCASTISSMETVVNGIKKFVYLKRWMDELEKLPCFEINRRGLERLKYFMSLYKPN

>BmGSTo1

MSEKHLQTGDVLPPYSGKLRVFAMRFCPYAERTVLTLNAKNIPYDLVFINLDQKPEWIFNFSPKGTVPALEYEPGKALFDSNIINVYLDEKYPEIPLQASDPLRRAQDKILVESFAPAQSAYYTAAFNAQALEPSMVETYHKGLEGLQKELETRSTKYLHGDEPGWVDYTLWPFLERFEALPLIGKAEFAIDQTKYERLVTYIEAMKNVPAVKSYFLAAETHAKFIESRAQGDANYNMLDTSAVCCMRPRKKKE

>BmGSTo2

MSAIKDSRNINFNIKHLRKGDPLPPFNGKLRVYNMRYCPYAQRTILALNAKQIDYEVVNIDLIDKPEWLTTKSAFAKVPAIEIAEDVTIYESLVTVEYLDEVYPKRPLLPQDPLKKALDKIIVEASAPIQSLFIKILKFSDTVNEEHVAAYHKALDFIQEQLKNRGTVFLDGSEPGYADYMIWPWFERLRAFAHDERVRLEPSKYSLLLEYIDNMLKDSAVSQYLIPLEILAKFHEAYTKKERPNYELLNECLKSF

>BmGSTo3

MTYFHSVNAGVIPPPALTDKLRLYHVDMNPYGHRVLLVLEAKRIKYEVYRLDPLRLPEWFRAKNPRLKIPVLEIPTDQGDRFLFESVVICDYLDEKYTRHTLHSHDPYVKAQDRLLIERFNELIKGSLECFDTNFAFGSEQIIQTLEIFEKELTNRGTNYFGGNRPGMLDYMVWPWVERLYLLRCVNDRKFVEKKSLFPNFADWGDQMQLDDIVKKHAHSPQEYFDYYKNARAHSMGYYL

>BmGSTo4

MVSPKINFNTKHLGKGDPLPPWSGKLRVYNMRLCPFAQRTILTLNAKQIDYEVINIDLVNKPEWLPTKSIFGKVPTIEVEDGVCICESLIIAEYLEEVYPEIPLISKDPIKKAYEKIIIEASEPIFVMYFKVMRTPDTINDETLMSYHKALTFFEGQLRNRGTRFLGGEKPGFADYMIWPWFERIQSMNDEKLKIKSAKFDLLVAYIENMYKDPAVSQYLLPKDVMDKLHAEYKTGKFEVQSIEDLL

>BmGSTs1

MPKVVYHYFACKALGESGRMLLAYGGQDFEDHRVLSADWPDFKPKTPFGQTPVLVIDGKQYAQSTAICRYLGRKYGLAGANDEEAFEIDQNVEFLHDIRAKAAAVYYEADEELKAKKHEDFSKNVYPDMLKKLNSIVEANKGHIAAGKLTWGDFVFTSMFDYLKTMLQIPDLEVQYPAFKKVLQSVLTQPKVKAFLDLGRPYEFEF

>BmGSTs2

MPNVKFYYFPVKALGESQRLLLAYGGQEFEDNRISSENWPEFKPKTPFGQMPVLEIDGKQYAQSTAICRYLGRKYGLAGANDEEAFEIDQNVEFLNDIRASAASVHYEKDEAVKAKKKAELEETKYPFFFEKLNEILTKNNGHIALGKLTWGDFVYAGMYDYLKAMLQKPDLEQKYPAFRKPIEAVLAIPKVKAYVDAAPRTEL

>BmGSTt1

MVLKLYYDLMSQPSRVLYILLKTMKYDFEPKYVNLRKAEHYSEDFTKVNRMQRVPVIDHNGFILTESIAILKYLSRENVIAESLYSKESKLQARIEEFLEWQHIGLRLHCAMYFRVVHMDPILTGRKSDEKTIQGYKRRMMMALDDFDTKWLGRGTAFIVGETPTVADLVAACELEQPRMAGFEPKDHFPNIAAWWPKVRDHFAPHYEDAHVILNKIINKMDRAANSKL

>BmGSTz1

MGKQPVLYSYWRSSCSWRVRIALNLKEIPYDIKAVSLIKGGGEQHCNEYREVNPMEQVPSLCIDGHTLIESLNIMHYLEETRPQRPLMPQDCFKRAKVREICEMIASGIQPLQNLIVLIYVGEEKKKEWSQHWITRGFRAIEKLLSTTAGKYCVGDEITLADCCLVPQVFNARRFHVDLRPFPIILRIDRELENHPAFRAAHPSSQPDCPPEVAK

>BmGSTz2

MVENRVILHAYWLSSCSWRVRAMLHAKSIPFEERPVDIVKTGKQLTEEYRAINPAQKVPALEIDGVTLVESTAIIQYIEDTRPEPKLMPDTALQRARMREICETIVSGIQPLQNFGLKKHLGTEEKFLSFTKYWTERGLQTLNDLLAKTSGAYCIGDQITLADICLVPQIYNGVSRHKLDLKTYPIVSKVYENLLKEELYQATHPKATKEKLKINL

>BmGSTu1

MVLKLYAVSDGPPSLSVRQALVALEVPFELINVDFGAGEHMTSDYALMNPQKEIPVLDDEGFYLSESNAILQYICDKYRPGSPLYPQDPKSRAIVNHRLCFNLSSYYANISAYTMAPIFFDYERTPLGLKKVHISLDVLETYLTRTNTSYAAANHLTIADFPLINSTMTLEAIDFDFSKYTKIHKWYNDFKVKYPDLWKISESAMKEIQHFAANPPDLTHLNHPIHPIRKIKN

>CsGSTd1

MRTLWSSFACISILIISVGNAVARSKNSKMPAQHIKLHYMPPSPPSRAVMMAAKVLGIDLELVLTNLMEGAHLTPEFLKMNPQHTIPTMDDNGFILWESRAILGYLVNAYGRDDTLYPKNPRLRALVDQRLYFDAGTLFSRYMTLYRPMLFEGAQMDETHAAKLNEAISWLNTMLEGRAFVAGDNLTIADISIIVTFTNLEAFDYDFSQYENVSKWFQRTKKALEPYGYNDIDVAGAQILANFLKN

>CsGSTd2

MPVDLYYVPGSAPCRTVLLAAKALKVDLNLKLVDLHHGEHLKPEYLKINPQHTVPTLVDDGFSVWESRAIITYLANKYAKGSPLYPEEPKARALVDQRLYFDIGTLYQRFSDYFYPQVFGGAPADKEKLAKVEDALKLLDQFLEGQKFAAGPALTLADLSLVASVSSMEASDVNFNKYNNIKRWYETVKMSAPNYEEANGKGLQAFKELVNNMMKK

>CsGSTd3

MAIDLYYTPGSAPCRLVLLVAAALNVELNHKLLNLRAGDQLAPDYLKINPQHTVPTIVDDGFALWESRAISRYLVTKYGEGGDLYPDDLHARAVVDQRLDFDLGTLYPRFAQYFYPQIFAGAQPDEGLYKKLGEALGFLDSFLDSSPGEYAAGPKLTLADLSLVATVSTIDAAGINLKPYLNVAKWFELVKNTAPGYQKANGEGIDMFKELIAKLKGKTEL

>CsGSTd4

MPIDLYYVPGSAPCRAVLLTAKALNLNLNLKLVDLHHGEHLKPEYIKLNPQHTVPTLVDDGFSIWESRAIITYLVNKYAKGSGLYPEEPKARALVDQRLYFDIGTLYQRFADYFYPQVFGGAPADKDKLAKIEDALQLLNTFLEGQKFCAGPNLTLADLSL

>CsGSTe1

MRTIVYQTDSSPPARAVKMILYILGLEFEIREMNPLLREQDAPEMRKKNPMRTVPFIEEGDFCLSDSHAIAIYLFEKYGNPEHEYLYPKDMRKRATVNQRLFFDCGILFQRLRSIMAPTYMGIMTEMPKEVILGIKNAYRMLEEYLSQSTYLADENMTIADVCAVATVSSLDGMYPVEEKRYPKTRRWLDTMYNKDFCRIANQPGCELHVAHLKFAMENNKLNKKAKL

>CsGSTe2

MVLTLYKKDTSPPSRAVLMTIHALQIPDVKLINVHLPDGEQFTEEYLKMNPQHTVPMLKDDDFIIWDSHAICGYLVSKYGNDDSLYPTDPKKRASVDQRLHFDSGVLFNTLKATVAPVLYTDDKDFRPENLAKFKECYDFMEKFLTKSWLAGDEVTIADICCVSTISSMNEIVPIDDKQYPKLTDWWRKCSEQEFYKKGNAPNILLFRELIKNKLAEKQRTV

>CsGSTe3

MPITIYKTDGSPPARAALMIIELLGLKVEKSEVMPLTGDHLKPEFVQKNPVHTIPFLEDGDFKLADSHAILTYLVSKYGAEKKAQLYPCDLEKRATVDQRMYMEATLVFPKIREIIATLFREKKKVPTEAQVEATNEVYGILDKYLEQTKFIAADHLTVADISLVSSISSLEGILPVDKFPKLSEWLETMKQNDWYQTANASGAKTFSDFVKQKSDAL

>CsGSTo1

MSEKHLQTGDVLPPYSGKLRLFAMRFCPYAERSVLVLNAKNLEYDLVFINLDHKPEWIFQFSPKGTVPALEYEQGKAIFDSNIINVYLDEKYPEKALQASDPLRRAEDKMIVENFSAAQSAYYTAAFNAQALQPSMVENYHKGLEGLQKELESRGTKFLHGAEAGLVDLTLWPFLERFLALPLLGKPEFAIDSTKYGLLTTYIEAMKAVPAVKSYYLAAETHAKFTESRAKGDPNYNMLDTSAVCCMRPRKKKE

>CsGSTo2

MWSWKVLKSTAPTSCKLCRNLTSAVKGKVCFNSKHLATGDQLPPYNGTLRVYNMRFCPFAQRTMLALIAKQIDYEVVNINLMNKPDWLFKKSAFGKVPALEIEENVTIFESLPTVEYLDEVFSNRPLLPKDSVQKNQMKMIVEASAGAIHNLFVKLVKYPEYITEGNITAFHKSLDFIQYQLISRNTKFLHGCEPGYADYMIWPWFERLRAADDARVKIDVETHATLIKYIDNMMQDSAVKEYLVPVNILREFHDGFQDTKGPNYDLLC

>CsGSTo3

MTYYEHRMAGAVPPPALTDKLRLYHVDMNPYGHRVMLILEAKKVKYEVYRLDPLRLPEWFKAKNPRLKIPVLEIPTEYGDRYLFESVVICDYLDEKYPRNPLHSRDPFVKAQDRLLIERFNELIKGSLECFDTNFVFGSEQIIQTLDIFEKELATRGTNYFGGNRPGMLDYMIWPWVERLYLLRCLNEKKFDGKRSLFPNFADWGDQMQLDDVVKKQSHSPQQYFEYYKNARAHSMGYYL

>CsGSTo4

MIASLVSLSGHVLVTSYKVLHTVVPALEYVSSYWTMGSIKVEFNTKHLEKGDPLPSYNGKLRVYNMRYCPWAHRAILALNAKELDYEIVNINTQNKPDWLPSKSAFGKVPALEIQDGVSIYESSVIVEYLDEVYPKRPLLSKDPVTRAFDKIIVEAFAPVTSLFFRAMTNPEGVTDDARTAYEKALHFVQEQLTLRGTKFFGGSQPGYVDYMIWPWFERITILEGLNGHAEIDPQKYKLLTDYIAEMFKDPAVSQYVVNKDVLLKFGDAYKKKLPINYDMLLEQ

>CsGSTs1

MPAVKLTYFPVKALGESTRLLLAYGGQEFEDFRINPETWPELKPKTPLGQLPVLEIDGKQYAQSLAIGRYLGRKYGLSGADIGEDFEIDQNVDFVNDIRARAALVHYETDEVLKEKKHEDFSKNMYPAMLKKLDEIITKNNGHLAAGKLTWGDFVLAGMFDYLKTMLRQPQLEQQYPSFKKLVDGVYALPQLAKYLAQAPKTEI

>CsGSTs2

MAKKLQYFNINGLGESIRYMLHYGGHKFEDIRYELKDWPIQSVKDSLPYGQLPLYEEGGKTLNQSLAIARYVASQSQLLPSDLWQQAVLDAVVHNIYDFWNKNKIAEYIRGDAETKAALKKQTFEEHIPFYFPRFEKELKTNKGHFAGKLTWADFILVGMIETANLFYGENIETNYPTIAALVKTIQNLPKVKEYIATRNPYTFK

>CsGSTt1

MSIKLYCDLMSQPSRTLYILLKTIKCDFETKLVDLRKGQHFSDEFTKINKIQRVPVIEHNGFILSESVAIVNYLSREGIIPDNLYPQDSKKRARVEEFLEWHHIGLRLPCSMYFRVKQLDPIITGKQPEAKTVAGYENRMLQSLDTFSTKWLNQGHDFLCGNTVTVADLFAACELEQPRMAGYDPKEHFPAIATWAKKVQQHFNPYYDEGHVIVNKVINKQKKMSSKI

>CsGSTz1

MAKPVLYSYWRSSCSWRVRIALNLKEIPYDIKAVSLIKGGGEQHCNEYREVNPMEQVPSLCIDGHTLVESLSIMHYLEETRPQRPLMPQDCFKRAKVREICEVISSGIQPLQNLIVLIHVGEEKKKEWAQHWITRGFRAVEKLLSSSAGKYCVGDDITLADCCLVPQVFNARRFHVDLRPFPIILRIDRELENHPAFRAAHPSAQPDCPPEVAK

>CsGSTu1

MVMKLYAVSDGPPSLAVRQALAHLQLPFELVSVDYGAGEHMTTEYALMNPQKEIPVLDDDGFFLSESNAILQYICDKYKPDSHLYPQEPKARAIVNHRLCFNLSTYYANISAYTMAPIFYDYDRTPLGLKKVHMALDVFETYLQRQGTQYAAADHLTIADFQLINTNMTLEAIGFDFSQYKRIHKWYNDFKKDYPDLWKISADAMKEIQHFAENPPDLTHLNHPIHPARKANK

>ApGSTd1

MTIDFYYASWSPPCRTVELVAYILKVKLNPIETIPSKGDTQKPEYKQLTPQHTIPTIVDNGFVLSESRAICKYLVEKYGSATGPYSKEQLYPKDLQKRAAIDHRIDFDLGSLYRRASDYFSPVFMTGHFGTAALPKLKAALEILDTYLAKTKWVAGPEVTLADIVVVVTISSLEIVGYELTNYPNILRWFKAAQTTLPGYNEANHKGRIEFKDYLFSKLKNN

>ApGSTd3

MTLIHSSSGEKDKKDIDLYFDPMSPPCRSVLLTLEALNLEINLINIRLPANRKRIENFLKNNYKNTKPPVPVIQDGDFVLLESHAIIVYLVREYGGKDDHSLYPDDPKIQAQVNQRLHFDNGTLYLAYKRQYIPWIYNRIAKTEDREKNIHEALEFLENVLKKSPWTAGDSMTVADFALVASISTFQVSGVDLNSYNNINKWLIKCANTMDGYENANQEGIAITKALLNYLEKFDDLNRPHLDNIYRSDKS

>ApGSTd7

MPIDFYYTPGSPPCRSVLLTAKALGLELNLKTLDLHHGEHMKPEFIKLNPQHCVPTLVDGDLVLWESRAIIVYLAQAYGKDDSLLPKDPKKQALVNQRLQFDVSTLYPAFSDQYYPWIFAGVPKSDDKEKKIHDALGFLDIFLGSSTWAAGDSVTVADLALVASISTIEAVGVDLSKYANVSKWFEKCKTTLVGYQEFNQKGIDGFKIMVANLTKK

>ApGSTd8

MFFKYYCRTELKLYYTPGSPPCRSVLLTAKALGLEFKLITVDLFKGDHMKPEFLKLNPQHSIPTLEIGEFIMWESRAIIVYLVQTYGKYNDLLFPKDKKKQARINQILQFDLGTLYPAFQRQYYPWLFGKELKTKDKEQKIHDALGFLETFLGSNDWVAGDSMTLADMALVASISTFEVAGVDLNKHVKVSKWLQKCKTTMEGYKDANQYGVDRFKALVEKKLADSVAQKEQGIDVGEIQEHNTVLVTNYKVFKATKTLLRAVKDHLIAEEFSEILKQLETAMEAIVN

>ApGSTd11

MTSTLIKYNVILIVLCIGITLIHNSSEATVKIENLKQDSTKEVNLYYDPFNPQCRSVLLTLGALDLELNLKRIYLFHSEQLPEDFRNINSLHTLPVMQDGDLVLSESNAIIVHLVRKYGGQDDHPLYPNNPKIQAKVNQGLHFNNSYFSQAFEIPHIFRGILKTAEVEDKIHEALNFLEEILEKSTWTAGNTITVADFALVASISTFEVVDFNLGNYQQIQNWLSKCKTTMASYDTANQEGIYELKSLLESKNGFQNKIKTEISLYYDPISPSCRSVLLTIKALNLEVNLKVIDLLADGTQEKDFLDINSLHTLPVMQDGELVLVESHAIIVYLVQVYGKKNDPLYPHDPTFQAQINQRLDFNNFYFYLAFEIQHDDKDDGRIPKIVGMDKIHKALKFLEEILKKSIWTAGNIMTVADFALVASISTFEAFDVDLGKYENIKNWLSLCKSVMPNYDWANQEGIYAVMAIR

>ApGSTd15

MPRIDYNTSSLYNIIRYYNTNPIVLTTTTVPSCRSVLLTIKALNLEVNLKVIDLLADGTQEKDFLDINSLHTLPVMQDGELVLVESHAIIVYLVQVYGKKNDPLYPHDPTFQAQINQRLDFNNFYFYLAFEIQHDDKDDGRIPKIVGMDKIHKALKFLEEILKKSIWTAGNIMTVADFALVASISTFEAFDVDLGKYENIKNWLSLCKSVMPNYDWANQEGIYAVMAIR

>ApGSTo1

MAIKHLSKDSVEPPKVPGSLRFYSMRFCPYAQRVQLILNAKGMPHDTVFIDLSDKPEWYLKIFPAGKVPALIYDDKFLSESLLLADFLDKQYPEPPLQASSPLQTILDKLVIESFGKVGTAFYKLIMTTKEIEKQNFDELVASLIPIETELVERGTKFFGGNKPNMVDYMIWPWFERLDAINPYSNGTFVIPFEDKFPRLAEWKSLMIADKAVAPYYITPEKHAEHFTKRKAGLPAYDI

>ApGSTs1

MTTYKLTYFNLTARAEQIRFLLSYLNVDFEDVRFEREQWPAIKPTMPFGKVPVLEIDGKTFNQSIAICRYLAKKAGLAGDDEWESLLIDVAVDNIYEIRQEIMNYYHEPNEEIKSKLRGPIVNDSIPFYIDRFENIVSENGGYFVNGKLSWPDLYFVSILDHIKSVIDVDLVDGRPHFTAFKHKVLAIPQIKSWIAKRPKSQ

>ApGSTs2

MAVYKLTYFNFPALAEPIRFLLSYLEIDFEDVRFEREQWPSIKPTMPFGKVPILEIDGKVLNQSAAISRYLSKKAGLAGSDEWESLLIDIAVDNVNDLRQAIALQVFDSNEESKAEKYVTLINETIPLYMNKFENTVVENDGYFVNGKLSLADIHFVAIIDFLSFLAKVDLLEGRPNLQAHKNKIFDIPQIKSWIAKRPAFSMKL

>ApGSTs3

MTYKLTYFNFTALGEPIRFLLSYLNIDFEDNRIEVEQWPSVKHTIPYGKLPLLEIDGKVLNQSTAICRYLAKKANLAGSDDWESLLIDIAVDNFQDFRLSIISYWYEQNEESKAAKYATLVNETIPYNMERFENIVIENNGYFVNGKLSWADLFFVGILDYLKFRSEIDLLKDRPNLQALREKVLAVPKIKSWIEKRPSTVDIA

>ApGSTs4

MSYKVTYFNITALAEPIRFLLSYLNIDFEDFRFEREQWPTLKPTMPFGKVPVLEIDGKVLNQSTAITRYLSKKAGLAGSDDWESMLIDIAVDNIHDLRQAIALYAYDSNEATKEARYAPLINETIPFYMDKFEKIVEENNGYFVNGKLSWADLFFVAILDYLNFMAKIDLLEGRPKLKALKEKVLEVPQIKAWVAKRPTDNP

>ApGSTs6

MTSYKLTYFNFTGLGEPIRFLLSYLDIDFEDNRIEIVQWPSVKHTMPYGTLPLLEIDGKVLNQSSAICRYLAKKANLAGSDDWESLLIDIAVDNYKDFALCLKSYWFEPNEETKAAKHIILVNETIPFYMEKFEKIVGENNGYFVNGKLSWADLFFVAVLDHLIYRLNIDLLKDRPNLQALQEKVLAVPKIKSWIERRPVSFDIA

>ApGSTt1

MAKLIYYHNLLSQPSRALYMFFKKAEVPFEGKVVDLLKGEQFTAEFEAINPFKKVPVVNANGFVLIESIAILRYICRTYNVADHWYPKDSVKQAQVDEYLEWQHTNTRADCALYYLHKVLWPVMNGKPVNEQRVAQLEKKMITTLDLIENVWLKNKTFLSGNEISISDIIAICEIDQTRIAGYNPYANRPNLSNWKMRTATYLSPYYEEANEILEMHVAKYNKKYGKLNSHI

>ApGSTt2

MVTLKFYYDFLSQPCRTLYIFMKKTKIPFEPKPVNLRQGEHLTKEFVSLNPFKKVPFIDDKGTVLIESVSILRYLCRTYNVADHWYPKDIQRQALVDQYLEWQHNNTRAHCTEYFRHKALWAAKNWPGSKY

>ApGSTm1

MISFEVDEVLFRTYVFYTAILVLKVLAMAPLTAKQRFAKMVFANPEDAKMNPKSKVKYDDADIERVRRAHLNDLENIPLFIIVCFGYLLTIPNVYIAINLIRLFVASRIIHTIVYAVVVLPQPARGLSWFAGFATTVYMAVQVILSFV

>ApGSTm2

MSDTSTMGLFMISNPVFECYAFYGSILILKMIMMSFLTALQRFRKKVFISPEDTAISKGGGEIRYDDPDVERVRRAHLNDLENIPIFLITGLLLVASKPQVMIANNLFRIYTFVRIMHTISYAVFVLPQPTRAILFIAGAVINIIMVAYVILSMHYF

>DmGSTd1

MVDFYYLPGSSPCRSVIMTAKAVGVELNKKLLNLQAGEHLKPEFLKINPQHTIPTLVDNGFALWESRAIQVYLVEKYGKTDSLYPKCPKKRAVINQRLYFDMGTLYQSFANYYYPQVFAKAPADPEAFKKIEAAFEFLNTFLEGQDYAAGDSLTVADIALVATVSTFEVAKFEISKYANVNRWYENAKKVTPGWEENWAGCLEFKKYFE

>DmGSTd2

MDFYYMPGGGGCRTVIMVAKALGLELNKKLLNTMEGEQLKPEFVKLNPQHTIPTLVDNGFSIWESRAIAVYLVEKYGKDDYLLPNDPKKRAVINQRLYFDMGTLYESFAKYYYPLFRTGKPGSDEDLKRIETAFGFLDTFLEGQEYVAGDQLTVADIAILSTVSTFEVSEFDFSKYSNVSRWYDNAKKVTPGWDENWEGLMAMKALFDARKLAAK

>DmGSTd3

MVGKALGLEFNKKIINTLKGEQMNPDFIKINPQHSIPTLVDNGFTIWESRAILVYLVEKYGKDDALYPKDIQKQAVINQRLYFDMALMYPTLANYYYKAFTTGQFGSEEDYKKVQETFDFLNTFLEGQDYVAGDQYTVADIAILANVSNFDVVGFDISKYPNVARWYDHVKKITPGWEENWAGALDVKKRIEEKQNAAK

>DmGSTd4

MDFYYSPRSSGSRTIIMVAKALGLELNKKQLRITEGEHLKPEFLKLNPQHTIPTLVDNGFAIWESRAIAVYLVEKYGKDDSLFPNDPQKRALINQRLYFDMGTLHDSFMKYYYPFIRTGQLGNAENYKKVEAAFEFLDIFLEGQDYVAGSQLTVADIAILSSVSTFEVVEFDISKYPNVARWYANAKKITPGWDENWKGLLQMKTMYEAQKASLK

>DmGSTd5

MDFYYSPRGSGCRTVIMVAKALGVKLNMKLLNTLEKDQLKPEFVKLNPQHTIPTLVDNGFSIWESRAIAVYLVEKYGKDDTLFPKDPKKQALVNQRLYFDMGTLYDSFAKYYYPLFHTGKPGSDEDFKKIESSFEYLNIFLEGQNYVAGDHLTVADIAILSTVSTFEIFDFDLNKYPNVARWYANAKKVTPGWEENWKGAVELKGVFDARQAAAKQ

>DmGSTd6

MDLYNMSGSPSTRAVMMTAKAVGVEFNSIQVNTFVGEQLEPWFVKINPQHTIPTLVDNLFVIWETRAIVVYLVEQYGKDDSLYPKDPQKQALINQRLYFDMGTLYDGIAKYFFPLLRTGKPGTQENLEKLNAAFDLLNNFLDGQDYVAGNQLSVADIVILATVSTTEMVDFDLKKFPNVDRWYKNAQKVTPGWDENLARIQSAKKFLAENLIEKL

>DmGSTd7

MPNLDLYNFPMAPASRAIQMVAKALGLELNSKLINTMEGDQLKPEFVRINPQHTIPTLVDNGFVIWESRAIAVYLVEKYGKPDSPLYPNDPQKRALINQRLYFDMGTLYDALTKYFFLIFRTGKFGDQEALDKVNSAFGFLNTFLEGQDFVAGSQLTVADIVILATVSTVEWFSFDLSKFPNVERWLKNAPKVTPGWEQNLESLQQGKKFLQDLQAAKEKEVKA

>DmGSTd8

MDFYYHPCSAPCRSVIMTAKALGVDLNMKLLKVMDGEQLKPEFVKLNPQHCIPTLVDDGFSIWESRAILIYLVEKYGADDSLYPSDPQKKAVVNQRLYFDMGTLFQSFVEAIYPQIRNNHPADPEAMQKVDSAFGHLDTFLEDQEYVAGDCLTIADIALLASVSTFEVVDFDIAQYPNVARWYENAKEVTPGWEENWDGVQLIKKLVQERNE

>DmGSTd9

MLDFYYMLYSAPCRSILMTARALGLELNKKQVDLDAGEHLKPEFVKINPQHTIPTLVDDGFAIWESRAILIYLAEKYDKDGSLYPKDPQQRAVINQRLFFDLSTLYQSYVYYYYPQLFEDVKKPADPDNLKKIDDAFAMFNTLLKGQQYAALNKLTLADFALLATVSTFEISEYDFGKYPEVVRWYDNAKKVIPGWEENWEGCEYYKKLYLGAILNKQ

>DmGSTd10

MDLYYRPGSAPCRSVLMTAKALGVEFDKKTIINTRAREQFTPEYLKINPQHTIPTLHDHGFALWESRAIMVYLVEKYGKDDKLFPKDVQKQALINQRLYFDMGTLYKSFSEYYYPQIFLKKPANEENYKKIEVAFEFLNTFLEGQTYSAGGDYSLADIAFLATVSTFDVAGFDFKRYANVARWYENAKKLTPGWEENWAGCQEFRKYFDN

>DmGSTe1

MSSSGIVLYGTDLSPCVRTVKLTLKVLNLDYEYKEVNLQAGEHLSEEYVKKNPQHTVPMLDDNGTFIWDSHAIAAYLVDKYAKSDELYPKDLAKRAIVNQRLFFDASVIYASIANVSRPFWINGVTEVPQEKLDAVHQGLKLLETFLGNSPYLAGDSLTLADLSTGPTVSAVPAAVDIDPATYPKVTAWLDRLNKLPYYKEINEAPAQSYVAFLRSKWTKLGDK

>DmGSTe2

MTDKIVLYGMDISPPVRACKMTLRALNLDYEYRELDLLAGDHCKDEFLKKNPQHTVPLLEDNGALIWDSHAIACYLVDKYAKSDELYPRDLVLRAQVNQRLYFDASILFMSLRNVSIPYFIRQVSLVPKEKVDNIKDAYGHLENFLGDNPYLTGSQLTIADFCCGATASSLAAVLDLDGERYPKVAAW

>DmGSTe3

MGKLTLYGIDGSPPVRAVLLTLRALNLDFDYKIVNLLEKEHLKPEFLKINPLHTVPVLDDNGFYLSDSHAINSYLVSKYGRNDSLYPKDLKKRAIVDQRLHYDSSVITSTGRAISFPLFWENKTEIPKARIDALEGVYKSLNLFLDSGNYLAGDNLTIADFHVIAAMTGNLVFLPVDANKYPDLAAWIQRIKELPYYEEANGSRAAQII

>DmGSTe4

MGKISLYGLDASPPTRACLLTLKALDLPFEFVFVNLFEKENFSEDFSKKNPQHTVPLLQDDDACIWDSHAIMAYLVEKYAPSDELYPKDLLQRAKVDQLMHFESGVIFESALRRLTRPVLFFGEPTLPRNQVDHILQVYDFVETFLDDHDFVAGDQLTIADFSIVSTITSIGVFLELDPAKYPKIAAWLERLKELPYYEEANGKGAAQFVELLRSKNFTIVS

>DmGSTe5

MVKLTLYGVNPSPPVRAVKLTLAALQLPYEFVNVNISGQEQLSEEYLKKNPEHTVPTLEDDGNYIWDSHAIIAYLVSKYADSDALYPRDLLQRAVVDQRLHFETGVVFANGIKAITKPLFFNGLNRIPKERYDAIVEIYDFVETFLAGHDYIAGDQLTIADFSLISSITSLVAFVEIDRLKYPRIIEWVRRLEKLPYYEEANAKGARELETILKSTNFTFAT

>DmGSTe6

MVKLTLYGLDPSPPVRAVKLTLAALNLTYEYVNVDIVARAQLSPEYLEKNPQHTVPTLEDDGHYIWDSHAIIAYLVSKYADSDALYPKDPLKRAVVDQRLHFESGVVFANGIRSISKSVLFQGQTKVPKERYDAIIEIYDFVETFLKGQDYIAGNQLTIADFSLVSSVASLEAFVALDTTKYPRIGAWIKKLEQLPYYEEANGKGVRQLVAIFKKTNFTFEA

>DmGSTe7

MPKLILYGLEASPPVRAVKLTLAALEVPYEFVEVNTRAKENFSEEFLKKNPQHTVPTLEDDGHYIWDSHAIIAYLVSKYGKTDSLYPKDLLQRAVVDQRLHFESGVIFANALRSITKPLFAGKQTMIPKERYDAIIEVYDFLEKFLAGNDYVAGNQLTIADFSIISTVSSLEVFVKVDTTKYPRIAAWFKRLQKLPYYEEANGNGARTFESFIREYNFTFASN

>DmGSTe8

MSKLILYGTEASPPVRAAKLTLAALGIPYEYVKINTLAKETLSPEFLRKNPQHTVPTLEDDGHFIWDSHAISAYLVSKYGQSDTLYPKDLLQRAVVDQRLHFESGVVFVNGLRGITKPLFATGQTTIPKERYDAVIEIYDFVETFLTGHDFIAGDQLTIADFSLITSITALAVFVVIDTVKYANITAWIKRIEELPYYEEACGKGARDLVTLLKKFNFTFST

>DmGSTe10

MANLILYGTESSPPVRAVLLTLRALQLDHEFHTLDMQAGDHLKPDMLRKNPQHTVPMLEDGESCIWDSHAIIGYLVNKYAQSDELYPKDPLKRAVVDQRLHFETGVLFHGIFKQLQRALFKENATEVPKDRLAELKDAYALLEQFLAENPYVAGPQLTIADFSIVATVSTLHLSYCPVDATKYPKLSAWLARISALPFYEEDNLRGARLLADKIRSKLPKQFDKLWQKAFEDIKSGAGKQ

>DmGSTs1

MADEAQAPPAEGAPPAEGEAPPPAEGAEGAVEGGEAAPPAEPAEPIKHSYTLFYFNVKALAEPLRYLFAYGNQEYEDVRVTRDEWPALKPTMPMGQMPVLEVDGKRVHQSISMARFLAKTVGLCGATPWEDLQIDIVVDTINDFRLSSEQFVSYEPEDEIKEKKLVTLNAEVIPFYLEKLEQTVKDNDGHLALGKLTWADVYFAGITDYMNYMVKRDLLEPYPALRGVVDAVNALEPIKAWIEKRPVTEV

>DmGSTm1

MDNGPMDATPTAAAFRLILLSKSNPVMGCYMFWTSLLVLKMLVMSLLTARQRMKTKTYANPEDLRLSRSTEVRFGDPNVERVRRAHRNDLENILPFLLMSLAYVASGPNPLTARLLIRIGASARLIHTVVYAIIPVPQPARALAFFTTFAITCFEAGYVLV

>DmGSTm3

MLNPELMSLENQVFRCYLGWSAILILKIFAAGIYTGLMRFFTATFANPEDLMSPKLKVKFDDPNVERVRRAHRNDLENILPFFAIGLLYVLTDPAAFLAINLFRAVGIARIVHTLVYAVVVVPQPSRALAFFVALGATVYMALQVIASAAF

>AgGSTd3

MDYYYSLISPPCQSAILVAKKLGITLNLKKTNVHDPVERDALTKLNPQHTIPTLVDNGHVVWESYAIVTYLVEVYGKDDTLYPKDPKVRSVVNQRLFFDIGTLYKQIIDIIHLVVKKEQPTDEQMEKLKKAMDLLEHFLTERSYAAADHLTVADICLLGSVTALNWLKYDLEPFPHIKGWVARVTGEIPDYAEFRKDVEEATKAYVASKK

>AgGSTd4

MDYYCNFVSPPSQSVILVAKKLGIKLNLRKMNIYDPVAMDTLSKLNPHHILPMLVDNGTVVFEPCAIVLYLVEMYAKNDALYPKDALVRCVVNQRLFFDVGTLYKQIYENVHVQMRNSQPSEKQVQRLQKAVDVLESFLYERSYTAADQLTVADICLLVTVNALTLWLGYELAPYPRIRDWLGRVVAELPGCVEFQREVEDATRAYVVNRKI

>AgGSTd5

MELYSDIVSPPCQNVLLVAKKLGIALNIKKTNIMDAADVAELTKVNPQHLIPTFVEDDGHVIWESYAIAIYLVEKYGQDDALYPKDPKVRSIVNQRLFFDIGTLYKNILANIDVLIEKQQPSAELRGKLEQALDLTEKFVTECRFVATDHLTLADIFMLGSITALEWFRYDLERYPGIRGWVERVTAQFPDYSDFHKEIREATKQYVATHCPHLEY

>AgGSTd6

MPRLDLYYNIISPPCRVVLLFAKWLKLELNLIELDVLKRDHYKPEFLKHYIPTLVDADGDVVVWESSAILIYLAERYGAADDDTLYPKDIALRAKVNQRLFYDIGTLMRSVTTYYHPILMGGEGKLEDFKKVQDAVGVLDSFLSASRWTAGDHITVADFAIAVTVAALDGLLNFDFSVYPNVHRWYEQCKRELVGYTDITKEAAQRTQAFLERFRAMRAADQQLLCEQQRTVRQRQKEDGTDDQQQQQQQQQQQQQQYTELQTQPRSGRTRDTAPDYARKPDESTRMATED

>AgGSTd7

MTPVLYYLPPSPPCRSVLLLAKMIGVELELKALNVMEGEQLKPDFVELNPQHCIPTLDDHGLVLWESRVILAYLVSAYGKDENLYPKDFRSRAIVDQRLHFDLGTLYQRVVDYYFPTIQLGAHLDQTKKAKLAEALGWFEAMLKQYQWSAANHFTIADIALCVTVSQIEAFQFDLHPYPRVRAWLQKCKDELQGHGYKEINETGAETLAGLFRSKLKQ

>AgGSTd10

MELYYNIVSPPCQSVLLVGKKLGITFDLKEVNPHLPEVREQLRKFNPQHTIPTFIEDGHVIWESYAIAIYLVEKYGNGDDALYPRDPKVRSVVNQRLFFDNGLMFKSAIEYVECILKKKLEPTEEMQQRLKKALGLLESFVKERAFVASDHLTIADICLLSSVTLLTGIKYDLATFPGITAWVARVTGELPDYGEFHKELYEKSMEYIKTL

>AgGSTd12

MDLYYHIRSPPCQPVVFLARHLGLEFNHIVTSIYDPADFEVLKKVNPQHTIPTLVDNGHILWESYAILIYLAEKYALDDSLYPKDVCERSIVHQRLFFDSGMFQNTTLQAVLSHLRNNPITDEHLAKVKRGVEIVEMYLTDSPYVAGQKLTIADFSIFVSFCSLDMMKYDLTAYPNVQRWFAKMGTHIPDLEPTRKTIEEELRALLQSMNK

>AgGSTd15

AKMELYSDIVSPPCQNVLLVAKKLGIALNTKKTNIMDAADVAELTKVNPQHLIPTFVEDDGHVIWESYAIAIYLVEKYGQDDALYPKDPKVRSIVNQRLFFDIGTLYKNILANIDVLIEKQQPSAELRGKLEQALDLTEKFVTECRFVAADHLTLADIFMLGSITALEWFRYDLERYPGIRGWVERVTAQFPDYSDFHKEIREATKQYVATHCPHLEY

>AgGSTd16

IHTMDLYYHIRSPPCQPVVFLARHLGLEFNHIVTSIYDPADFEVLKKVNPQHTIPTLVDNGHILWESYAILIYLAEKYALDDSLYPKDVCERSIVHQRLFFDSGMFQNTTLQALLSHLRNNPITDEHLAKVKRGVEIVEMYLTDSPYVAGQKLTIADFSIFVSFCSLDMMKYDLTAYPNVQRWFAKMGTHIPDLEPT

>AgGSTd17

MRNSQPSEKQVQRLQKAVDVLESFLYERSYTAADQLTVADICLLVTVNALTLWLGYELAPYPRIRDWLGRVVAEIPGCAEFQREVEDATRAYVVNRKI

>AgGSTe1

MPKPVLYTVHLSPPCRAVELTAKALGLELERKLVNLLAGENLTPEFLKLNPKHTIPVLDDNGTIISESHAIMIYLVRKYGQGEGKDALYPTDIVEQARVNEALHFESGVLFARLRFITELVFFARKPEIPEDRIEYVRTAYRLLEDSLQSDYVAGSRMTIADLSCISSVASMVGFIPMERSEFPRVHGWIERMKQLPYYEEINGAGATELAEFIVDMLAKNAKL

>AgGSTe2

MSNLVLYTLHLSPPCRAVELTAKALGLELEQKTINLLTGDHLKPEFVKLNPQHTIPVLDDNGTIITESHAIMIYLVTKYGKDDSLYPKDPVKQARVNSALHFESGVLFARMRFIFERILFFGKSDIPEDRVEYVQKSYELLEDTLVDDFVAGPTMTIADFSCISTISSIMGVVPLEQSKHPRIYAWIDRLKQLPYYEEANGGGGTDLGKFVLAKKEENAKA

>AgGSTe4

MPNIKLYTAKLSPPGRSVELTAKALGLELDIVPINLLAQEHLTEAFRKLNPQHTIPLIDDNGTIVWDSHAINVYLVSKYGKPEGDSLYPSDVVQRAKVNAALHFDSGVLFARFRFYLEPILYYGATETPQEKIDNLYRAYELLNDTLVDEYIVGNEMTLADLSCIASIASMHAIFPIDAGKYPRLAGWVKRLAKLPYYEATNRAGAEELAQLYRAKLEQNRTNAK

>AgGSTe5

MATNPIIKLYTAKLSPPGRAVELTAKLLGLSLDIVPINLLAGDHRTDEFLRLNPQHTIPVIDDGGVIVRDSHAIIIYLVQKYGKDGQTLYPEDPIARAKVNAGLHFDSGVLFSRLRFYFEPILYEGSAEVPQDKIDYMKKGYELLNDALVEDYIAGSSLTLADVSCIATIATMEEFFPMDRSRYPALVAWIERLSRTLPEYDQLNQEGAVEFAEICESLRLKNGASVAAK

>AgGSTe6

MSSKPVLYTHTISPAGRAVELTVKALNLDVDVREMNVFKGQHMSDEFKKLNPVQTIPTLDDNGFVLWDSHAIMIYLARRYGAESGLYTEEYEQQARINAALFFESSILFARLRFCTDNLTVLGKSAIPEENLQRALEGLQRLERMLQSEYVAGDQLTIADLSCVSSVATLHLMLKPSAEEFPKTFAWMERLSKLPYYGEVMGRGLKAAGELMQTLGSKNSGGGGDGN

>AgGSTe7

MEPSRLVLYTNRKSPPCRAVKLTARALGIELVEKEMTLLRGDKLMEEFLKVNPQQTIPVLDDGGIVITASHAIMIYLVCKYGRDDGLYPSELVRRARVHTALHLEAGVIFSRLSFLFEPVIYSGKSYFHSDRIEHIRKAYRLLEDSLVDQYMVGESLTIADFSCISSIATLVGVVPLDESKFPKSTAWMRRMQELPYYEEANGTGALELAEFVLGKKEANASQFL

>AgGSTo1

MSNGKHLAKGSSPPSLPDDGKLRLYSMRFCPYAQRVHLMLDAKKIPYHAIYINLSEKPEWYLEKNPLGKVPALEIPGKEGVTLYESLVLSDYIEEAYSAQQRKLYPADPFSKAQDRILIERFAGSVIGPYYRILFAADGIPPGAITEFGAGLDIFEKELKARGTPYFGGDKPGMIDYMIWPWCERVDLLKFALGDKYELDKERFGKLLQWRELMEKDDAVKQSFISTEDHTKFLQSRKNGENNYDILA

>AgGSTs1

LSSSSISRSSLKCNIMPDYKVYYFNVKALGEPLRFLLSYGNLPFDDVRITREEWPALKPTMPMGQMPVLEVDGKKVHQSVAMSRYLANQVGLAGADDWENLMIDTVVDTVNDFRLKIAIVAYEPDDMVKEKKMVTLNNEVIPFYLTKLNVIAKENNGHLVLGKPTWADVYFAGILDYLNYLTKTNLLENFPNLQEVVQKVLDNENVKAYIAKRPITEV

>AgGSTt1

MSKNLKYYYDLMSQPSRALWIFLEKTKLPYEKCLINLGKGEHLTEEFKAINRFQKVPCITDSQIKLAESVAIFRYLCREYQVPDHWYPADSRRQALVDEYLEWQHHNTRATCAIYFQYVWLRPRMFGTKVDPKQAEKYRGQMEGTLDFIEREYLGSGARFIAGDEITVADLLAACEIEQPRMAGYDPCEGRPNLTQWMARVRESTNPYYDQAHKLVNKFAQDTASKAKL

>AgGSTt2

MSRSVKLYYDLMSQPSRALYIFLSTNKIPFDRCPIALRKMQHKTDEYRRQVNRYGKVPCIVDGSFRLAESVAIYRYLCREFPTDGHWYPSDTVRQARVDEYLSWQHLNLRADVSLYFFHVWLNPLLGKEPDAGKTERLRRRLDGVLNFFDQELLSAGSGQAFLAGDRISIADLSAACEIEQAKIAGYDPCEGRPALASWLTAVRERTNPYYDEAHKYVYRLSPDHIVTPVVAEDE

>AgGSTz1

MSLSAMSKPILYSYWRSSCSWRVRIALNLKEIPYDIKPISLIKSGGEQHCNEYREVNPMEQVPALQIDGHTLIESVSIMYYLEETRPQRPLMPQDVLKRAKVREICEVIASGVQPLQNLIVLIHVGEEKKKEWAQHWITRGFRAIEKLLSTSAGKFCVGDEITLADCCLVPQVFNARRFHVDLRPYPIILRIDRELEGHPAFRAAHPSNQPDCPPEAAK

>AgGSTm1

MTTLLQNVNEEVFRTYVFWTAVLVVKMLAMSVLTGRQRFRKKVFANPEDIQPSKKGAQPKFDDPDVERVRRAHRNDLENILPFFAIGLLYMLTNPEPFIAINLFRAVAIARIVHTLVYAVVVIPQPARGLSWAIAYFATAYMAVKTALFFL

>AgGSTm2

MASPFDSINSEAYKAYVFWSAVLVAKMLLMALLTAIQRFKNKAFASPEDTRVISKKLVPKYDDPDVERVRRAHQNDLENILPFFVIGFLYLLTNPAPWLAINLYRLVAASRILHTIVYAVVVIPQPARFLAFVGAMMPTAYMTLQTILYFML

>AgGSTm3

MSLVFGQVEPAVFQAYAFWAAVLGLKMLLMSVLTGLKRGSKKVFSNPEDVKPGGKVAYDDQDVERVRRAHRNDMENILPYFIIGFLYMFTNPSVTVATNLFRLVAVVRISHTVFHVLVPVHKFRGMSWAIGFFTTAFMGIQIVLHFL

>AgGSTu1

MKLYAVSDGPPSLAVRMALEALNIPYEHVSVDYGKAEHLTAEYEKMNPQKEIPVLDDDGFFLSESNAILQYLCEKYAPTSDLYPNDPKDRALVNHRLCFNLAFLYPQISAYVMAPIFFDYERTAIGLKKLHLALAAFETYLQRTGTRYAAGSGLTIADFPLVSSVMCLEAIGFGLGERYPKVQAWYDGFKQAHPSLWAIAAKGMEEIAEFEKNPPDLTGMVHPIHPIRKPAAK

>TcGSTd1

MPIDLYYLPGSAPCRAVLLAAKAVGVELNLKLTDLMKGEHLTPEFIKINPQHTIPTMVDNGFALWESRAIMTYLADQYGKNDALYPKDPKKRALVDQRLYFDIGTLYARFADYYYPVIFGGAEYEPAKLEKIKDAFKFLEIFLEGQDFVAGNQLTLADLSLLATVTTFEAVNFDLSPYKNVVNWLARAKAAAPGYEEANGKGAVIFKQMVENLTKK

>TcGSTo1

MSVTHLTTGSQQPLKVDGKLRLYSAEFCPYAQRVRLVLKAKNIPHDIVNISLSHKPEWYSKIHPEEKVPALDTGTKIIIESLDIVEFLDEQYPKNPLYPLEPEAKKRDQELVKKLSPLSDALFKCVLSHKVKTLGQCMAELVPQFEIFETELAARGSPFFGGRTPGMVDYLLWPTCERLGVLAIAYGEQLPFDENQLKFLKKWNKAMFENPICQETYHEPEEHWIVVQQKLNFLKTK

>TcGSTo2

MPQPHLTTGSPQPPKIEGKLRLYSMEYCPYAHRVRLVLNAKNIPHDIVNINLINQPEWYFKIHPQGYVPALDTGSQIVIESLHICDFLDEKYPSPPLFPQDPASKQRDKDLLKKIQPMHGVFLRCISLNENKSLEEWATEFVPHLETFETELSNRGTTFFGGEKPGMVDYMLWPWGERVGTIVIAHGQQLPFASDQFPLLRKWRKAMRGDPVCDGLYYGPEKYWKIIQIKFRKAPPEYDNV

>TcGSTo3

MASPHLTTGSQQPPKIEGKLRLYSMQFCPYAQRARLVLKAKNIPHDIVNINLINKPEWYTKVHPEGKVPALDTGSKIVVESLDIADFLDAEYPNNNPLYSSDKNRDKELIKKIAPITDLFYKCVAKTENKSLEEWAKAFVPHLEVFERDLAARGTTFFGGDKPGMVDYMLWPWGERAGTIAIAHGAQLPFGSDQFPCLRKWRKAMREDKICSEIYNGPEKFWKCVEMKLKNLPPDYDSI

>TcGSTs1

MSPLYKLTYFATPGRAEAIRFLFSYAAVEFEDVRIAYEDWPALKNQTPFGFLPMLEHEGKKAHQSVAIMRYVAKQVKLAGNDDWEDLEIDATVDTLRDCSSKFHPLRLETDEEKKKALLEQLFKETVPYFMRRFEALVQKNNGYLALGRLTWADLYFVASIAAGVKQYTDIDIIKEYKTLAELRTKVLENPRIKKYLENQPKLKMAPAYKLTYFEYTGLAEVSRFLMKFGGIDFEDCRIKMEEWPQLKAKFPFGQVPVLEYKGKIACQSLAIARYLAKQVKLSGNDDWENLEIDATIDTINDLRMKLATWWFEADEAKKKLIVENFKKDNLSYYLPRLEAIVKKNKGFLAVGRLTWADFYWATISPVFDMVTGIDALADYPELKAARERVNALPAVKKWIDERPNN

>TcGSTs2

MSPLYKLTYFATPGRAEAIRFLFSYAAVEFEDVRIAYEDWPALKNQTPFGFLPMLEHEGKKAHQSVAIMRYVAKQVKLAGNDDWEDLEIDATVDTLRDCSSKFHPLRLETDEEKKKALLEQLFKETVPYFMRRFEALVQKNNGYLALGRLTWADLYFVASIAAGVKQYTDIDIIKEYKTLAELRTKVLENPRIKKYLENQPKLKMAPAYKLTYFEYTGLAEVSRFLMKFGGIDFEDCRIKMEEWPQLKAKFPFGQVPVLEYKGKIACQSLAIARYLAKQVKLSGNDDWENLEIDATIDTINDLRMKLATWWFEADEAKKKLIVENFKKDNLSYYLPRLEAIVKKNKGFLAVGRLTWADFYWATISPVFDMVTGIDALADYPELKAARERVNALPAVKKWIDERPNN

>TcGSTs3

MAPAYKLTYFDGRGLAETSRFLMKYGGIDFEDCRIKREEWPQWKPKFPFGQVPVLEHKGKVVGQSIAIARYLAKQVKLVGNDDWENLEIDAIVDTINDLRMKHAAWFYEPDEAKKKVIIENIKKDTLPYYLPRLEEIVKKNKGFLAVGRLTWADFYWATVSQVFDVVNGIDTLANYPELKAARERVNSLPAIKKWIEVRPKTDF

>TcGSTs4

MAPAYKLTYFPVEALAEPIRFLLNYGGIEFEDHRFDRENWPQLKPNMPFGQVPILEYNGKVAHQSVAMARFFAKKVKLVGNDDWEDLEIDAIVDTISDLRQKIALYHYEQNEAVKESRKEPLFKETIPYYLQRLDAIVKANNGHLAVGKLTWADLFFVALLKYLCFMCGSDIIADYPNLVALKKSVLEIPAIKNWVEKRPKSDM

>TcGSTs5

MSPNYKLIYFNARGRAEHIRFIFAYAGVEYEDERIPREKWPEIKKRTPFGMLPVLEIDGKAVAQSNAVARYLARQYGLAGRNEWEALQCDVLVDTLGDLKQVLAQFRMEQDPIKKEEKKARLMKETIPFYLSKFEKILSENNGFSVGSEITWCDFVFAVSLENFEHIFGKAALDQYPALKALKIKVYSIPSINAWVSKRPATES

>TcGSTs6

MAPAYKLTYFDGRGLAETSRFIMKYGGIDFEDCRIKREDWPQIKSKYPFGQLPVLEHNGKTVNQSHSIARYLAKQVKLAGNDDWENLEIDAIVDTFNDLRLKIVAYFYEQDEEKKKTILENLNKDVFPQYLTRFEEIVKKNKGYFALGRLTWADFCWATVSPGFDMITKVDTIANYPELKAVRDKVNSLPAIKKWIEQRPKTDF

>TcGSTs7

MAPAYKLTYFDARGLAETSRFLMTYGGIDFEDCRIRREEWPLWKPKFPFGQVPVLEHRGKVVGQSTAIARYLAKQVKLVGNDDLENLEIDAVVETINDLRLKHAAWFYEPDEAKKRIIIENIKKDTLPYYLPRLEEIVTGNKGFLALGRLTWADFYWTTVSQIFDVVTGTDTLANYPELKAARERVNSLPAIRRWIEMRPKTDF

>TcGSTt1

MTLKLYLDFLSQPSRALYIFFKINKVPFELSQVALRKGEHLSEEFKTNLNRFQKVPFIHDGDFRLTESVAIIRYVSKVHNIDNNWYPKETKAQARVDEYLEWQHNNTRAFCALYFQRKWLFPLLTGRQTSPETMQKYEDNMLACLDQIENIWLADTPYLCGDRISVADIFAACEIEQPRVAGFDPIKGRPVLSAWMNRVRSEASPFYEEAHAVLNKLAEKGGKAKL

>TcGSTz1

MSGKPLLYSYWRSSCSWRVRIALNLKEIPYDIKPVSLIKTGGEQHTNEFREVNPMEQVPALHIDGVTLVESLSILAYLEETRPQRPLLPHDVVKRAKVREICEVIASGIQPLQNLVVLIHVGEEKKNEWAQHWINRGFRAVEKLLSASAGKYCVGDEITLADCCLIPQVFNARRFHVDLRPFPIILRIDRELENHPAFRAAHPSNQPDCPPEIANTNCFSPDPYV

>TcGSTm1

MAQVTDFSTLLESPVFRAYLFYSAILVVKMMIMSPMTGMMRFRYKAFANPEDGASLKVKPRTDDNVERVRRAHLNDLENISLFFVIGFIYVLTNPAVAWATLLFRIYTAARFMHTLVYAIFVVPQPARALAWVTGFVITGYMALTSIVHFL

>TcGSTm2

MDPHLKSLILENPVFRSYLFYSSILALKMMLMTLLTIRQRLLNNALVSQEDAAFLGGTVCWTNEKVERVRRGHRNDMESIYLFLLVAFAYIWTDPEPVWADYLFLTFTIARILHTVVYNVIVVPQPARGLAWLVGYLITGYMAIKTYTAFK

>TcGSTm3

MVNTTSSAGPPDSVLTLNNPAFGVYLISACLLVLKMMGMSLLTIYNRFKYKAFICPEDAKWLQGQVVSNDTVERVRRAHQNDLENIPIFLAAAFAYLWTQPPAWLAWVLYLGFTILRALHTIVYTLIVLPQPTRALLWVAGYLLTGYMAVHAALHVFIYLIM

>TcGSTm4

MDVHQLRMLVTENPVFRSYMFYTAILTLKMMFMSLLTIRQRVMHNSFVSEEDAMYLKGMVSRTNEHVERVRRGHRNDMENIYLFFVIGFAYTWTDPSPFFANLLFFIFTVSRLIHTCVYTVVIMPQPIRGRAWLVGFLVTGYMAIRTLLHFC

>TcGSTu1

MPITLYSVSDGPPSLAVRQCLKMLNVEFNLVNVDFGLGEHMTEEYAKKNPQKEIPVLDDNGFYLGESNAILQYLADKYGKDDKLYPKDLQTRAIVNHRLCFNLSTYYRYISEHVMAPIFFDYARTPLTLKKVHIALDNFNTYLQRRGTKYAAADHITIADFQLVTATMCLEAINFDFSSYPLVTKWYATYKKEYPELWAIVEGGMKEISTFEKNPPDLSHMNHPIHPIRRH
